# Nutritional Determinants of Type 2 Diabetes Mellitus in the European Union: A Systematic Review

**DOI:** 10.3390/nu17223507

**Published:** 2025-11-09

**Authors:** Daniela Alejandra Díaz-Benavides, Abdu Nafan Aisul Muhlis, Ghenwa Chamouni, Rita Charles, Digafe Tsegaye Nigatu, Jomana Ben Khadra, Frederico Epalanga Albano Israel, Bashar Shehab, Gabriella Laila Tarek, Aidai Sharshekeeva, Nasser Gammoh, Tulu Tefera Habte, Niyati Chandrika, F. K. Alshakhshir, Nour Mahrouseh, Carlos Alexandre Soares Andrade, Szabolcs Lovas, Orsolya Varga

**Affiliations:** 1 Department of Public Health and Epidemiology, Faculty of Medicine, University of Debrecen, 4028 Debrecen, Hungary; diazdaniela@mailbox.unideb.hu (D.A.D.-B.); nafan.abdu@med.unideb.hu (A.N.A.M.); frederico.israel@med.unideb.hu (F.E.A.I.); asharshekeeva@mailbox.unideb.hu (A.S.); tulutefera@gmail.com (T.T.H.); nour.mahrouseh@med.unideb.hu (N.M.); lovas.szabolcs@med.unideb.hu (S.L.); 2 Doctoral School of Health Sciences, University of Debrecen, 4032 Debrecen, Hungary; 3 School of Public Health, Yekatit 12 Hospital Medical College, Addis Ababa 257, Ethiopia; digts1@gmail.com; 4 Department of Periodontology, Oro-Dental and Implant Surgery Faculty of Medicine, Universityof Liège, CHU of Liège, 4000 Liège, Belgium

**Keywords:** type 2 diabetes, nutrition, dietary patterns, ultra-processed foods, Mediterranean diet, Europe

## Abstract

**Background/Objectives**: Type 2 diabetes mellitus (T2DM) represents a growing public health burden in the European Union (EU), largely driven by modifiable lifestyle factors such as diet. This systematic review aimed to synthesize observational evidence on the associations between nutritional exposures and incident T2DM across the EU-28, with a focus on regional heterogeneity and implications for EU-level nutrition policy. **Methods**: The review followed PRISMA 2020 guidelines and was registered in PROSPERO (CRD42020219994). Searches were conducted in different databases (PubMed, Embase, Scopus, Web of Science) identifying 23,437 records, from which 104 observational studies were included. Eligible studies involved adults (≥18 years) without T2DM at baseline and assessed dietary exposures in relation to T2DM incidence. Data extraction and methodological quality assessment were performed in duplicate using the NHLBI tool in Covidence. **Results**: Most included studies were cohort studies (77.9%), primarily conducted in Western, Northern, and Southern Europe. Diets characterized by high consumption of whole and minimally processed foods, such as fruits, vegetables, legumes, whole grains, and fermented dairy, consistently showed associations with lower T2DM risk. In contrast, high intake of red and processed meats, sugar-sweetened beverages, and ultra-processed foods was linked to higher risk. Adherence to Mediterranean or plant-based dietary patterns was associated with lower risk, whereas high animal-protein dietary patterns were detrimental. **Conclusions**: Nutritional determinants play a decisive role in shaping T2DM risk in the EU. Evidence supports prioritizing dietary patterns rich in plant-based and minimally processed foods while reducing ultra-processed and meat-based products. Tailored region-specific policies are needed to address the T2DM epidemic and guide effective prevention strategies.

## 1. Introduction

Type 2 diabetes mellitus (T2DM) represents a major public health challenge across the European Union (EU), with rising incidence driven largely by modifiable lifestyle factors, including diet quality, physical activity, and weight management [1]. By 2030, an estimated 38 million adults in Europe are projected to live with the disease, approximately 90% of whom will have T2DM [2]. The financial burden of T2DM is substantial, estimated at EUR 150 billion in 2019 across the EU, primarily due to complications associated with the disease [3].

The ageing population contributes significantly to this growing burden, as older adults are more susceptible to T2DM and its complications, with global prevalence estimates reaching 20% among those aged 65–79 years [4]. Moreover, shifts in European lifestyles and the adoption of energy-dense diets high in sugar and fat have exacerbated adverse metabolic outcomes. The increased consumption of fast food, driven by convenience, affordability and accessibility, has been strongly linked to higher T2DM prevalence [5,6]. Existing evidence indicates that certain dietary behaviors, such as high intake of ultra-processed foods (UPFs) [7], red and processed meats [8], and sugar-sweetened beverages (SSBs [9], are positively associated with T2DM risk, whereas greater consumption of legumes, whole grains, and adherence to Mediterranean-type dietary patterns are linked to a lower risk [10,11].

Improving dietary patterns by reducing added sugars and unhealthy fats plays a key role in preventing T2DM. For example, an intervention promoting adherence to the Mediterranean diet lowered T2DM incidence by 52% compared with a control group [12]. However, substantial differences exist among European nations in terms of food consumption patterns, food composition regulations, and public health priorities, which in turn influence nutritional exposures and disease outcomes. These differences can modify the observed associations between diet and T2DM, underscoring the importance of region-specific evidence synthesis [13,14].

Moreover, improving dietary behavior depends heavily on food literacy and general health literacy [15]. Low literacy levels are associated with a higher risk of developing T2DM, highlighting the need to strengthen these skills across populations [16].

Considering the substantial heterogeneity in dietary habits, food environments, and T2DM burden across Europe, region-specific analyses are essential to inform culturally and contextually relevant prevention strategies. Previous reviews have often pooled data from multiple word regions, limiting the applicability of their findings to the European context and overlooking regional nuances in diet–disease relationships [17,18,19].

Therefore, this systematic review (1) restricts synthesis to EU-28 cohorts (2003–May 2023), (2) interprets the findings within key EU policy frameworks (e.g., Farm-to-Fork, CAP, WHO European Food & Nutrition Action Plan, EU4Health), and (3) examines regional heterogeneity within Europe (Northern, Western, Southern, and multinational studies) to offer EU-relevant policy implications.

## 2. Materials and Methods

This systematic review was conducted in accordance with the Preferred Reporting Items for Systematic Reviews and Meta-Analyses (PRISMA) 2020 guidelines [20], and it follows a methodological approach similar to that described by Andrade, C.A.S. (2025) [21]. The protocol was registered in the International Prospective Register of Systematic Reviews (PROSPERO) under the identifier CRD42020219994.

### 2.1. PECOS Strategy and Research Question

The review was structured using the PECOS strategy (P = Participants, E = Exposure, C = Comparison, O = Outcomes, S = Types of Study) [22] to define the scope and inclusion criteria.

- Population: Adults (≥18 years) without diagnosed T2DM, may include prediabetes at baseline, residing in the EU-28.
- Exposure: Dietary and nutritional exposures, including dietary patterns, specific foods, macronutrient and micronutrient intake, or other nutrition-related behaviors.
- Control: Populations with lower or no exposure to the nutritional factor of interest.
- Outcome: Incidence of T2DM, as measured by clinical diagnosis, fasting glucose, HbA1c, or self-report confirmed by medical records.
- Study Design: Analytical peer-reviewed observational studies, including prospective and retrospective cohort studies, case–control studies, and nested case–control studies.

The main research question was: “Among adults in the EU-28 countries, what associations exist between dietary or nutritional exposures and the incidence of T2DM?”

Secondary research questions included:

- What is the association between adherence to specific dietary patterns and the incidence of T2DM among adults without diagnosed T2DM in the EU-28?
- What is the regional impact on dietary patterns adherence and its association with T2DM risk?

### 2.2. Eligibility Criteria

Studies were eligible [21] for inclusion if they (1) were original, peer-reviewed, observational prospective or retrospective, such as cohort studies, case–control studies, and nested case–control studies; (2) had full-text availability; (3) reported data on at least one of the EU-28 member states; (4) included adult populations (≥18 years) free from T2DM at baseline but could include individuals with prediabetes; (5) examined associations between dietary or nutritional exposures, such as dietary patterns, specific foods, macronutrient or micronutrient intake, or nutrition-related behaviors, and the incidence of T2DM; (6) reported effect estimates such as relative risk (RR), odds ratio (OR), hazard ratio (HR), or incidence rates; and (7) were published in English or Spanish.

Studies were excluded [21] if they (1) used an ineligible study design, including non-original research (e.g., reviews, commentaries, editorials, or study protocols), interventional studies (e.g., randomized controlled trials), or cross-sectional, or descriptive, or laboratory-based, animal, or in vitro studies; (2) did not include a clear dietary or nutritional exposure; (3) lacked data on T2DM incidence or did not report sufficient statistical information (e.g., effect estimates or follow-up information); (4) combined data from the EU and non-EU countries without providing the EU-specific results; (5) presented redundant information (e.g., preprints later published). In this case only the peer-reviewed version was included.

### 2.3. Search Strategy, Study Selection, and Data Extraction

With a focus on the EU-28, the search strategy for this systematic review started on 1 January 2023 and concluded on 1 March 2023 [21]. It was developed around three core keyword themes: T2DM, nutritional exposures (including dietary intake, food groups, and macronutrients), and observational study designs. Comprehensive search strategies were constructed using a combination of subject headings (e.g., MeSH terms) and keywords, adapted to each electronic database: PubMed, Embase, Scopus, and Web of Science (the customized search strategies for each database are shown in the Supplementary File S1 adapted from previous publications [21]). The search results that were included in this review covered studies published between January 2003 and May 2023.

The search strategy [21] was based on the PECOS framework, and the most appropriate MeSH terms and Boolean operators were used:

“(((observational study) OR (clinical trial) OR (clinicaltrial) OR (“Clinical Studies as Topic”) OR (Controlled Before–After Studies) OR (Case–Control Studies) OR (“Cohort Studies”)) AND (type 2 diabetes mellitus)) AND (((“diet, food, and nutrition” OR “Risk Factors”) OR “Life Style” OR “Exercise” OR “Leisure Activities” OR “Preventive Health Services” OR “Communication” OR “Health Behavior” OR “Risk Reduction Behavior” OR “Dietary Supplements” OR “primary prevention” OR “prevention and control” OR “Social Determinants of Health”) AND (Austria OR Belgium OR Bulgaria OR Croatia OR Cyprus OR Czech Republic OR Denmark OR Estonia OR Finland OR France OR Germany OR Greece OR Hungary OR Ireland OR Italy OR Latvia OR Lithuania OR Luxembourg OR Malta OR Netherlands OR Poland OR Portugal OR Romania OR Slovakia OR Slovenia OR Spain OR Sweden OR “United Kingdom” OR Europe OR “European Union”))”.

Search results were exported in RIS format and imported into the Covidence systematic review platform (www.covidence.org) to facilitate deduplication and screening.

Fifteen trained reviewers working in pairs conducted screening independently. A pilot calibration phase was conducted using a random sample of thirty studies. Meetings were held to resolve conflicts and standardize the screening process. Disagreements at any stage of screening were resolved through consensus meetings with the two reviewers involved in the disagreement and a third, more experienced reviewer. Four reviewers mediated the disagreements in meetings.

Studies were initially screened on title and abstract using three options in Covidence, “yes,” “maybe,” and “no”, followed by a full-text screening using “yes” and “no”, based on specific exclusion criteria applied at both screening stages in this specific order: (1) full text not available; (2) irrelevant article type (e.g., reviews, editorials, conference abstracts); (3) data reported from outside the EU-28 countries; (4) inappropriate study design (e.g., interventional studies, simulation models, descriptive or cross-sectional studies); (5) off-topic content (not addressing T2DM); (6) irrelevant exposure (e.g., genetic factors, non-modifiable risks); (7) irrelevant outcome (e.g., lacking risk estimates or reporting only intermediate biomarkers); (8) T2DM at baseline.

To improve search completeness, citation tracking (snowballing) was conducted for all included articles. Reference lists were screened manually, and forward citation searches were performed using Google Scholar and Scopus. Articles identified through snowballing were subjected to the same inclusion and exclusion criteria.

Data extraction was carried out independently in duplicate using a standardized form developed in Covidence, based on the JBI Manual for Evidence Synthesis [23]. Extracted variables included study characteristics (first author, year, study design, country, cohort name), participant characteristics (age range, sex, baseline health status), exposure information (type of nutritional exposure, dietary assessment methods), outcome definitions (diagnostic criteria for incident T2DM, follow-up duration), effect measures (hazard ratios, odds ratios, relative risks with 95% CI) and covariates adjusted for in multivariable models.

### 2.4. Data Synthesis

Dietary exposures were categorized according to a meta-analysis by Schwingshackl, L., et al. (2017) [17]. These categories were consolidated into seven thematic groups to guide synthesis:

- Whole & Minimally Processed Foods: nutrient-dense, low-processed foods commonly recommended for metabolic health.
  Includes: Dairy products, eggs, fruits, vegetables, cereals and cereal products, fish and seafood.
- Animal-Based & Protein-Rich Foods.
  Focus: High-protein foods with potentially divergent health effects depending on processing and source.
  Includes: Red meat and meat products, animal fats and oils.
- Processed & Discretionary Foods.
  Focus: Highly processed, energy-dense products typically associated with poor metabolic outcomes.
  Includes: Sugars and confectionery, bakery products, snacks and desserts, alcoholic beverages, and condiments.
- Composite & Special-Purpose Foods.
  Focus: Foods developed for specific populations or health contexts, including clinical trials or supplementation.
  Includes: Infant foods, food supplements, composite meals, and products for special nutritional use.
- Sugar-sweetened beverages.
  Focus: All drinkable products, categorized by potential metabolic effects.
  Includes: Non-alcoholic beverages (e.g., coffee, tea, soft drinks) and alcoholic beverages.
- Carbohydrates.
  Focus: Quantitative and qualitative aspects of carbohydrate intake.
  Includes: Glycemic index/load, fiber, sugars, and starches.
- Dietary Patterns.
  Focus: Holistic eating approaches such as the Mediterranean diet, Western diet, or other predefined food-based patterns.

This categorization enabled a structured comparison of the associations between different types of exposures and T2DM incidence, facilitating a meaningful synthesis despite methodological diversity among the included studies. Each included study was summarized based on the direction (inverse, positive, or inconclusive), magnitude, and consistency of association between the nutritional exposure and the incidence of T2DM. Studies were grouped by exposure type, and regional comparisons were noted when possible.

To facilitate regional analysis, the European countries included in the studies were categorized into three main geographical regions based on conventional classifications. Western Europe comprised the UK, the Netherlands, France, and Germany. Northern Europe included Sweden, Denmark, Finland, and Lithuania. Southern Europe encompassed Spain, Italy, and Greece. In addition, a separate category labeled “Multinational” was used for studies that involved multiple countries spanning more than one region or that included countries not typically grouped within the three primary regions (e.g., multi-country studies including combinations of Western, Northern, and Southern European nations).

### 2.5. Methodological Quality Assessment

It was performed in parallel with data extraction within the Covidence platform [21]. Reviewers completed the National Institute of Health National Heart, Lung, and Blood Institute (NHLBI) quality assessment tool for observational cohort and cross-sectional studies [24], which is specifically designed to evaluate internal validity and risk of bias based on 14 core domains. The tool was adapted as needed based on each study design [24].

Studies were evaluated independently by two reviewers, and discrepancies were resolved in a consensus meeting involving a third reviewer. The meeting included a consensus on the data extraction form and the quality assessment form at the same time. Quality was rated based on the presence or absence of issues in four highly critical domains (items 2, 6, 9, and 11), as well as overall risk of bias across all items, assigning studies as low, moderate or high methodological quality.

- Low Methodological Quality:
  Critical domain questions: One or more “no” answers, or one “no” answer combined with one “cannot determine/not reported”, or two or more “cannot determine/not reported” answers.
  Non-critical domain questions: Three or more “no” answers, or four or more “cannot determine/not reported”, or one “cannot determine/not reported” response combined with two “no” answers.
- Moderate Methodological Quality:
  Critical domain questions: One “no” answer and one “cannot determine/not reported” answer.
  Non-critical domain questions: Two “no” answers, or three “cannot determine/not reported”, or one “cannot determine/not reported” response combined with one “no” answer.
- High Methodological Quality:
  Critical domain questions: No “no” answers and no “cannot determine/not reported”.
  Non-critical domain questions: One “no” answer and at most two “cannot determine/not reported” responses.

The interrater reliability was automatically calculated within Covidence using Cohen’s kappa coefficient (κ), which was 0.62 for the interrater reliability assessment, to assess consistency across reviewers [21]. Quality ratings were used to support the interpretation of findings but not to exclude studies from synthesis. To evaluate the influence of study quality on overall conclusions, a narrative sensitivity analysis was conducted. Associations were re-examined after excluding studies rated as “low quality” according to the NHLBI tool [24], to determine whether study quality altered the direction or magnitude of effects.

## 3. Results

Given the heterogeneity in effect measures and covariate adjustments across the included studies, a meta-analysis was not feasible. Therefore, we present a structured narrative synthesis, reporting measure-specific (HR/OR/RR/IRR) ranges for each exposure category, together with study counts and typical follow-up times.

### 3.1. Study Selection

**Figure 1:**
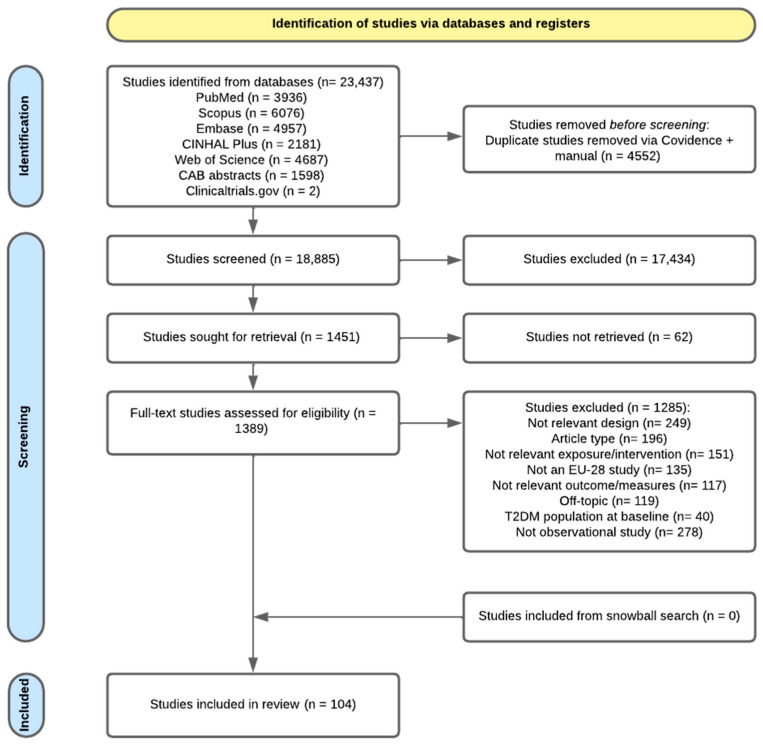
Preferred Reporting Items for Systematic Reviews and Meta-Analyses (PRISMA 2020) flow diagram.

The process of study selection and details of screening are presented in the PRISMA 2020 flow diagram (Figure 1).

A total of 23,437 records were identified through comprehensive database searches, with contributions from PubMed (n = 3936), Scopus (n = 6076), Embase (n = 4957), CINHAL Plus (n = 2181), Web of Science (n = 4687), CAB abstracts (n = 1598), and Clinicaltrials.gov (n = 2). After removing 4552 duplicates using Covidence and manual verification, 18,885 records were retained for title and abstract screening. Of these, 17,434 records were excluded based on predefined inclusion criteria, which were sought for retrieval. The remaining 1389 full-text articles were assessed for eligibility, with a result of 104 observational studies included in the review.

### 3.2. Study Characteristics

From the total of 104 studies included in this systematic review, the majority were classified as cohort studies (n = 81; 77.9%), with additional designs including case–cohort (n = 17; 16.3%), case–control (n = 5; 4.8%), and case-referent (n = 1; 1.0%).

Regarding population age categories, most studies focused on middle-aged adults (35–55 years) (n = 47; 45.2%) or older adults (55+ years) (n = 52; 50%). In 5 studies (4.8%), the age category could not be precisely determined based on available data.

In terms of outcome measures, most studies reported HRs (n = 77; 74.0%), followed by ORs (n = 17; 16.3%), and RRs (n = 7; 6.7%). Only a few studies reported risk difference (RD) (n = 1; 1.0%), rate ratios (n = 1; 1.0%), or incidence rate ratios (IRR) (n = 1; 1.0%).

**Figure 2:**
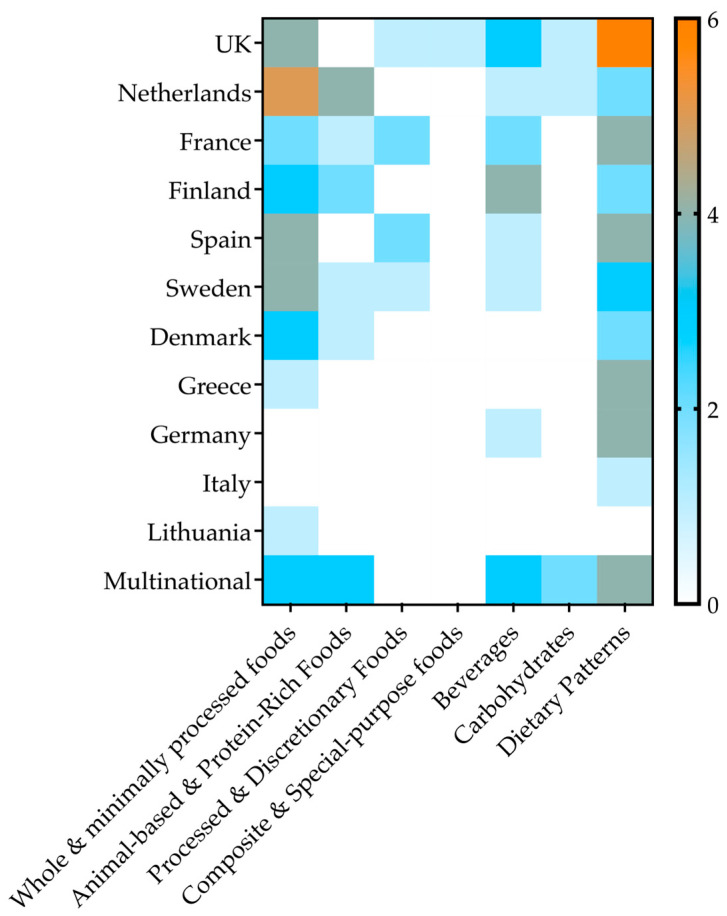
Distribution of included studies by dietary exposure category across European countries.

Geographically, the studies were primarily conducted in Western Europe (n = 44; 42.3%), Northern Europe (n = 28; 26.9%), and Southern Europe (n = 17; 16.3%). A portion of the studies (n = 15; 14.4%) were multinational investigations (e.g., studies including several European countries). The distribution of studies by dietary exposure category across countries is presented in Figure 2.

### 3.3. Quality Assessment

The methodological quality of the included observational studies was evaluated using the NHLBI quality assessment tool for cohort and cross-sectional studies [24]. Overall, 43.8% of the studies were rated as being of high quality (n = 46), 22.9% as moderate quality (n = 24), and 33.3% as low quality (n = 35), due to issues such as unclear exposure measurement or incomplete follow-up. Restricting the synthesis to high-quality studies did not materially change the direction of associations. Protective relationships for Mediterranean, plant-based, and whole or minimally processed dietary patterns, as well as for coffee and dietary fiber, were maintained. Harmful associations for UPF, processed and red meats, sugar-sweetened and artificially sweetened beverages, and Western-type diets also persisted. Effect ranges became narrower, and some extreme values were attenuated. These consistent trends are further detailed in the following section.

**Figure 3:**
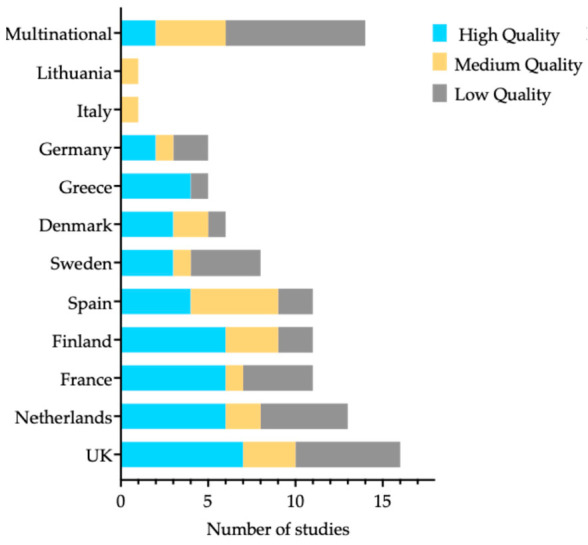
NHLBI quality assessment of included studies by country of publication.

The detailed quality assessment for each included study is available in Supplementary Table S1, and the distribution of study quality by country is presented in Figure 3.

### 3.4. Main Findings

Associations between diet and T2DM risk were examined across EU population and are summarized by protective and harmful dietary factors, providing an integrated overview of evidence from European cohorts relevant to current EU public health priorities on diet and chronic disease prevention.

Overall, inverse associations with T2DM risk were strongest for Mediterranean, plant-based, and whole-food dietary patterns, as well as for higher consumption of fruits, vegetables, legumes, whole grains, fermented dairy products, coffee, and tea. Adherence to Mediterranean or Healthy Nordic dietary indexes was associated with lower T2DM incidence across multiple cohorts [25,26,27,28,29,30,31]. Similarly, plant-rich diets emphasizing minimally processed foods showed consistent inverse associations [10,32,33,34,35,36,37]. Substituting red or processed meat with yogurt, cheese, or whole grains was linked with lower risk in Nordic and Western European cohorts [37]. Higher fiber intake and favorable carbohydrate quality were also associated with lower T2DM risk [38,39], and moderate consumption of fermented dairy, particularly yogurt, was inversely associated with T2DM risk [40,41].

Conversely, harmful associations were observed for diets characterized by higher intake of processed and red meats, UPF, SSB, and Western-type dietary patterns. Elevated consumption of processed or red meats was consistently linked to higher T2DM risk across Western and Southern European studies [42,43,44,45], higher SSB and UPF intake was associated with higher risk [46,47,48,49]. Western-type and high-fat dietary patterns were linked to higher risk [50,51,52].

When analyses were restricted to high-quality studies, the direction of associations remained consistent. Lower risk linked to Mediterranean, plant-based, and whole-food dietary patterns, and higher risk linked to UPF, SSB, and Western-type diets, were maintained, though effect ranges became narrower and extreme values were attenuated. This indicates that study quality influenced the magnitude but not the direction of associations, supporting the robustness of the overall findings.

Comprehensive numeric effect ranges, country-level coverage, and follow-up durations are presented in Table 1 and detailed study-level results in Supplementary Table S1.

**Figure 4:**
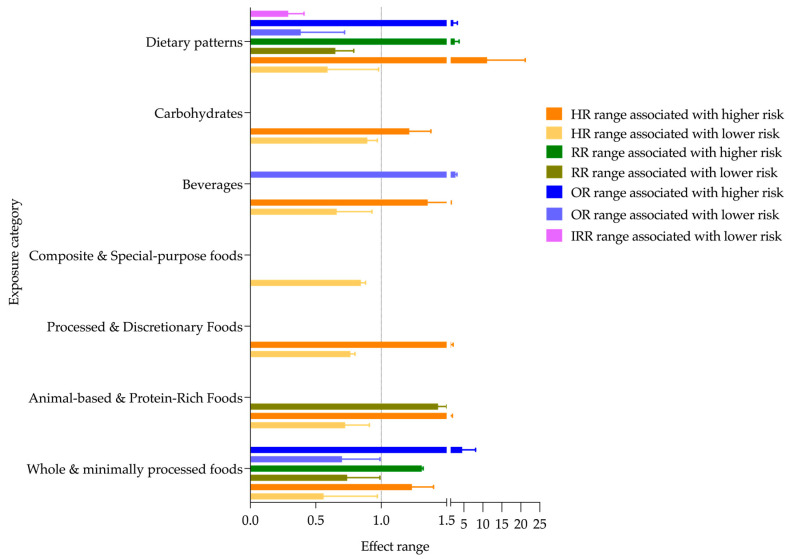
Ranges of HR, OR, RR, and IRR by exposure category, showing associations with T2DM risk. The dashed vertical line at 1 represents no effect; values to the left indicate association with lower risk of T2DM, and values to the right indicate a higher risk. Abbreviations: HR = Hazard ratio; RR = Relative risk; OR = Odds ratio; IRR = Incidence rate ratio.

To provide a visual overview of the magnitude and direction of associations between dietary exposures and T2DM risk, Figure 4 presents the ranges of HR, OR, RR, and IRR for each food group and dietary pattern, categorized by whether they were associated with lower or higher risk of the disease. This figure complements Table 1 by illustrating the variability of reported effect sizes across European cohorts.

## 4. Discussion

Our findings underline that the nutritional determinants of T2DM are not uniformly distributed across Europe; instead, they reflect marked geographical heterogeneity in both dietary exposures and their metabolic consequences. By explicitly considering regional differences in food culture and consumption patterns, this review advances the evidence base for developing context-specific prevention strategies, rather than relying on generalized global estimates.

These regional variations highlight the need for context-specific EU-level nutrition policies and closer alignment between scientific evidence with public health decision making.

In this systematic review, which included 104 observational studies from the EU-28, most of them being prospective cohort studies, we identified consistent patterns in the association between diet and T2DM risk. Our findings align with current European dietary recommendations [123], indicating that higher intake of whole and minimally processed foods, such as legumes, whole grains, fruits, vegetables, and fermented dairy products, is associated with a lower risk of T2DM. In contrast, dietary patterns characterized by greater consumption of red and processed meats, SSB, and UPF are linked to higher risk.

These findings are biologically plausible since dietary patterns rich in fiber, phytochemicals, and unsaturated fats (e.g., Mediterranean-type diets) promote insulin sensitivity and glycemic homeostasis [124], whereas high consumption of UPF and added sugars increases caloric density, adiposity, and inflammatory processes related to insulin resistance [125].

However, the magnitude of these effects varies between studies, and for some food categories (e.g., specific types of preparation or degrees of fermentation), the evidence remains heterogeneous. This suggests that food quality (e.g., fried fish vs. baked fish) and substitution patterns play a critical role [40,41,50,58].

Across the EU-28 countries, studies examining whole and minimally processed foods generally support their association with lower T2DM risk. Yet, inconsistencies emerge when regional dietary habits and metabolic profiles are considered. For instance, legumes, whole grains, fruits, and fermented dairy products are frequently associated with lower risk [10,32,33,35,36,40,41,57,58,59,65], although results sometimes diverge due to differences in food processing methods, cultural consumption patterns, or underlying population health [42,50,53,55,126,127].

Evidence on animal-based and protein-rich foods reveals a more polarized picture. While most studies indicate that high intake of red and processed meats is associated with higher T2DM risk [43,44,45,72,73,74,75], findings on alternative protein sources, such as fish, eggs, or dairy, are less consistent [37,69,70,71].

These discrepancies are likely shaped by regional dietary traditions, food quality, and substitution patterns. For example, the impact of dairy products appears to vary significantly depending on fat content, fermentation, and cultural dietary context [70].

The relationship between UPF, refined sugars, and T2DM risk is consistent. A broad consensus indicates that higher intake of these foods is associated with higher risk [46,47,77,78]. However, the health impacts of dietary components cannot be fully understood without considering cultural, culinary, and metabolic contexts [68].

Beverage consumption also illustrates the complexity of diet–disease relationships. Sugar-sweetened beverages are uniformly associated with elevated T2DM risk [38,48,49,81,88,90,92], whereas unsweetened coffee and tea frequently show associations with lower risk [80,82,83,84,85,86,87,88]. Nonetheless, preparation methods (e.g., filtered vs. boiled coffee), regional consumption habits, and accompanying dietary patterns substantially modify these associations.

The literature on carbohydrates further emphasizes the importance of quality and context. Not all carbohydrates exert the same metabolic effects: naturally occurring sugars (e.g., in fruits) and high-fiber whole grains differ substantially from added sugars and refined starches [39,93,94]. These findings call for more precise classifications in future research and dietary guidelines that distinguish between carbohydrate subtypes, food matrices, and glycemic impacts.

Dietary patterns offer the clearest consensus. Adherence to Mediterranean-style [26,27,28,96,97,104,114,115] or plant-forward diets [100,116] is consistently associated with lower T2DM risk across multiple EU countries. Nevertheless, the magnitude of inverse associations varies, with discrepancies noted among subgroups based on psychological, behavioral, or socioeconomic factors. Moreover, national dietary scores [103,119] may inadequately capture cultural variation within and between countries, limiting their applicability in diverse populations.

Regional contrasts revealed broadly consistent associations across Europe, but with varying strength and direction by geographic area. Cohorts from Northern Europe [31,32,33,37,41,43,48,50,54,57,62,63,65,66,69,70,76,82,83,84,92,99,102,103,111,119,120,128] showed the most homogeneous protective patterns, particularly for whole grains, berries, and Healthy Nordic dietary indexes, reflecting long-standing national nutrition policies and higher baseline dietary quality. Evidence from harmonized cohort analyses such as the EPIC calibration study confirms that Northern dietary models tend to feature more regular meal patterns, greater consumption of whole-grain and fish-based foods, and stronger adherence to public dietary guidance [129]. These culturally embedded habits, combined with coordinated policy frameworks such as the Nordic Nutrition Recommendations, may partly explain the consistency of protective associations [130].

In contrast, Southern Europe [10,27,28,34,36,40,60,77,81,96,97,104,112,114,115,116,131] displayed wider heterogeneity, with both direct and inverse associations observed across subgroups. Traditional Mediterranean dietary patterns, historically protective due to their high content of fruits, vegetables, legumes, and olive oil, have undergone gradual erosion linked to socioeconomic transitions, urbanization, and increasing consumption of animal products and convenience foods [132]. The resulting westernization of diets and decline in home-cooked meals may have weakened the protective effects once characteristic of Mediterranean populations. Western European studies [25,26,29,30,35,39,42,44,46,47,49,51,52,53,55,56,61,64,68,72,73,74,78,79,80,85,86,87,90,91,94,95,98,100,101,109,110,113,117,118,121,122,126,127,133] showed moderate inverse or neutral associations, potentially reflecting mixed dietary exposures, higher consumption of UPF, and variations in adjustment models.

Central and Eastern Europe remain notably underrepresented, with an absence of prospective cohort data. This evidence gap constrains region-specific inference and limits the capacity to design context-sensitive prevention strategies.

Overall, these patterns suggest that while healthy and minimally processed dietary exposures are associated with lower T2DM risk across Europe, regional dietary habits, food availability, and socioeconomic factors may modify their effects. Future prospective cohorts in underrepresented regions, particularly Central and Eastern Europe, are urgently needed to confirm these differences and improve the generalizability of evidence for dietary-prevention policies.

These findings also reveal how policy environments shape nutritional outcomes. Addressing these requires EU-level action that integrates dietary evidence into agricultural, fiscal, and health policy. The regional evidence summarized in this review directly supports the EU Farm-to-Fork Strategy [134] goal of enabling sustainable, health-promoting diets. The inverse associations observed for whole and minimally processed foods, alongside the positive associations of UPF and sugary products, underscore the need for upstream interventions. These include fiscal and regulatory measures such as SSB taxation and interpretive front-of-pack labelling (FOPL) schemes, consistent with the WHO European Food and Nutrition Action Plan 2015–2020 [135]. Moreover, the Common Agricultural Policy (CAP 2023–2027) [136] could incentivize production and affordability of healthier, minimally processed foods, such as legumes, whole grains, and fresh produce, particularly in regions where protective dietary patterns are less prevalent.

Because effect sizes and food matrices vary by region, EU-level interventions should combine common EU-wide measures (e.g., FOPL, SSB taxation) with targeted investments and surveillance in Central and Eastern Europe, through research funding, cohort establishment, and capacity building, to fill the evidence gap. By recognizing regional heterogeneity not as a limitation but as an opportunity for tailored policy design, the EU can align dietary, health, and sustainability goals across its diverse population.

This review has several limitations. The evidence base is uneven across Europe, with limited or no data from Central and Eastern European countries, which restricts the generalizability of findings and highlights the need for region-specific research to inform context-sensitive public health strategies. Most included studies relied on observational designs and self-reported dietary intake, raising concerns about residual confounding and measurement error. Substantial methodological heterogeneity was also present, including differences in exposure definitions (food groups vs. nutrients), dietary assessment methods, T2DM diagnostic criteria, and follow-up durations.

Although several included studies were classified as high or moderate quality, some were rated as low quality, primarily due to insufficient reporting or handling of follow-up procedures, such as incomplete follow-up and limited strategies to address attrition. However, restricting the synthesis to high-quality studies did not materially change the direction of the main associations, suggesting that study quality influenced the precision rather than the overall consistency of findings. These methodological shortcomings may nonetheless introduce bias and reduce the reliability of less robust evidence. Such heterogeneity complicated direct comparisons and precluded a formal meta-analysis; therefore, a narrative synthesis was applied. Finally, the possibility of publication bias cannot be excluded, as studies reporting null associations are less likely to be published.

The practical implications of this systematic review extend to the design of public policies that promote diets based on whole and minimally processed foods while reducing exposure to UPF, SSB, and red and processed meats. Beyond general measures such as food labeling, fiscal tools (e.g., taxation and subsidies), and restrictions on marketing, our findings emphasize the importance of tailoring interventions to regional dietary patterns within Europe.

For instance, aligning agricultural and trade policies under the CAP with health objectives could help shift food environments toward healthier options, while adapting national food-based dietary guidelines to local cultural and consumption contexts would enhance their effectiveness [134]. Such context-specific policies are essential for addressing geographical disparities and ensuring equitable progress in the prevention of T2DM across EU Member States [135].

In this way, nutrition policies that are responsive to regional dietary patterns can not only advance progress toward SDG 3.4 by reducing premature mortality from non-communicable diseases but also contribute to SDG 2 by fostering healthier and more sustainable food systems in Europe [137].

In summary, while many consistent patterns emerge across the EU, such as the harms of processed foods and the benefits of plant-based diets, regional differences in food systems, preparation methods, and cultural practices shape the association between diet and T2DM risk.

### 4.1. Policy Recommendations (EU Level)

- Farm-to-Fork/CAP alignment: Use CAP instruments to shift support toward the production, storage, and distribution of legumes, whole grains, and fruit/vegetables, and phase down measures that favor highly processed inputs.
- Front-of-pack labelling & marketing restrictions: Adopt a single interpretive front-of-pack label across MS and set EU minimum standards to restrict marketing of HFSS/UPF to children, allowing MS to go further.
- Fiscal measures (EU guidance; national adoption): Issue EU guidance for evidence-informed SSB excise taxes and encourage earmarking revenues for healthy food subsidies/vouchers in low-income regions; ensure state-aid compatibility and equity monitoring.
- Surveillance & research investment: Fund longitudinal cohorts and dietary assessment standardization in Central & Eastern Europe to address the regional evidence gap through EU4Health/Horizon Europe and with ECDC/EFSA coordination.

**Figure 5:**
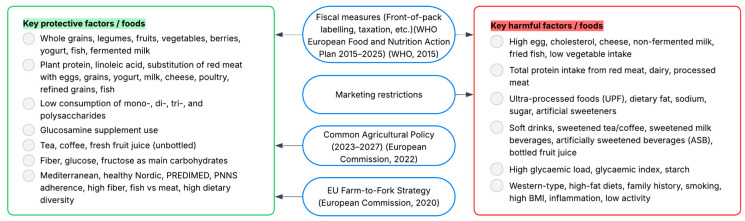
Conceptual flowchart summarizing protective and harmful dietary patterns and their relevance to EU-level policy frameworks for T2DM prevention [134,135,136].

A visual summary of these relationships is provided in Figure 5, which illustrates how dietary risk and protective factors identified in this review correspond to actionable EU policy levers for T2DM prevention.

### 4.2. Future Research

There is a clear need for prospective cohort studies and harmonized dietary assessment in Central and Eastern Europe. We recommend targeted funding (EU4Health, Horizon Europe) to establish longitudinal cohorts, standardize food classification (including UPF definitions), and collect high-quality outcome ascertainment. Comparative studies using harmonized protocols would allow robust region-specific effect estimates and inform tailored policy interventions.

## 5. Conclusions

By integrating nutritional epidemiology with the EU-specific regulatory frameworks, this review provides a foundation for clinicians, policymakers, and researchers seeking to address the burden of T2DM through evidence-based dietary interventions. Across 104 observational studies, high consumption of whole minimally processed foods was consistently associated with a lower risk of T2DM, whereas diets rich in red and processed meats, SSB, and UPF were linked to higher risk. These associations were broadly consistent across Europe but varied in strength by region, with Northern cohorts showing the most homogeneous protective patterns and Southern and Western regions displaying greater heterogeneity. Although several included studies were of lower quality, sensitivity analyses restricting the synthesis to high-quality studies confirmed the same directional associations, reinforcing the robustness of the main findings. Nonetheless, methodological diversity and uneven regional representation, particularly the scarcity of data from Central and Eastern Europe, warrant cautious interpretation. Future prospective studies with standardized dietary measures and regionally balanced cohorts are needed to guide effective, context-specific public health and policy actions across the EU.

## Figures and Tables

**Table 1:** Summary of findings by exposure category, number of studies, countries, effect range, factors and follow-up range.

Exposure Category	No. Studies	Countries	Effect Range		Factors	Follow-Up Range
			Including All Studies	Including Just High-Quality Studies		
Whole & minimally processed foods	30	Finland; France; Spain; Germany; Italy; UK; Sweden; Lithuania; Netherlands; Denmark; Greece	HR 0.15–0.97 [10,32,35,40,41,53,54,55,56,57,58,59] RR 0.49–0.99 [33] OR 0.41–0.99 [34,60,61,62]	Direction maintained HR 0.15–0.97 [32,36,41,55,57,58] RR 0.49–0.99 [33] OR 0.40–0.60 [60]	Berry, whole grains, legumes, white rice, egg, yogurt, fish, fruits, vegetables, fermented milk intake	1.8–20 years
			HR 1.07–1.40 [41,42,50,55,57,62] RR 1.30–1.32 [51] OR 1.03–8.07 [34,61,62,63,64,65,66,67]	Direction maintained HR 1.07–1.40 [41,42,55,57] OR 1.132.08 [65]	High egg, cholesterol, cheese and non-fermented milk intake, fried fish and shellfish, low dairy and vegetable consumption, metabolic factors (BMI ≥ 30, IFG, IGT)	
Animal-based & Protein-Rich Foods	12	Netherlands; Finland; Sweden; France; Denmark; Germany, Italy; Spain; UK; Greece; Norway	HR 0.54–0.91 [68,69,70,71]	Direction maintained HR 0.80–0.95 [68]	Plant protein, linoleic acid, SFA 15:0 and 17:0 intake, substitution of red meat (with eggs, whole grains, yogurt, milk, cheese, poultry, refined grains, fish)	3.4–19.3 years
			HR 1.04–1.97 [44,45,72,73,74,75] RR 1.37–1.50 [43]	Direction maintained HR 1.30–1.87 [44,72] RR 1.37–1.50 [43]	Total protein intake (red meat, fish, dairy, processed meat)	
Processed & Discretionary Foods	6	Sweden; Spain; France; UK	HR 0.73–0.80 [76]	ND	Low consumption of mono-, di-, tri- and polysaccharides	5.4–18.4 years
			HR 1.05–2.17 [46,47,76,77,78]	Direction maintained HR 1.12–2.17 [46,47,77,78]	UPF, dietary fat, sodium, and sugar intake, frequent use of artificial sweeteners	
Composite & Special-purpose foods	1	UK	HR 0.81–0.88 [79]	ND	Glucosamine supplement use	8.1 years
Beverages	16	France; Spain; Sweden; Germany; Finland; UK; Netherlands; Denmark; Italy	HR 0.39–0.93 [49,80,81,82,83,84,85,86,87,88,89]	Direction maintained HR 0.64 (≥7 cups/day coffee) [84]	Tea, coffee, fresh fruit juice (as substitution for the bottled)	6.9–13.4 years
			HR 1.03–1.68 [38,49,88,89,90,91,92] OR 2.42–3.17 [48]	Direction maintained HR 1.18–1.68 [90,91,92]	Soft drinks, sweetened tea/coffee, sweetened milk beverages, artificially sweetened beverages (ASB), and bottled fruit juice	
Carbohydrates	4	UK; Netherlands; Sweden; France; Italy; Germany	HR 0.82–0.97 [39,93,94]	Direction maintained HR 0.82–0.85 [39]	Fiber, glucose and fructose as main carbohydrates	6.3–12 years
			HR 1.05–1.38 [93,94]	ND	Glycemic load, glycemic index and starch	
Dietary Patterns	36	UK; Spain; Greece; Germany; Sweden; France; Netherlands; Denmark; Finland; Italy	HR 0.20–0.98 [25,26,27,30,95,96,97,98,99,100,101,102,103,104,105,106,107,108] RR 0.51–0.79 [31,109] OR 0.05–0.72 [28,29,110,111] IRR 0.17–0.41 [112]	Direction maintained HR 0.41–0.96 [25,26,27,95,99,102,106] RR 0.72–0.79 [109] OR 0.03–0.73 [28,29,111]	Mediterranean diet, healthy Nordic food index, PREDIMED dietary pattern, PNNS guideline adherence, healthy lifestyle score, fiber adherence, fish vs. meat, high dietary diversity	3.5–23 years [25,26,27,28,30,31,52,95,96,97,98,99,100,101,102,103,104,106,110,112,113,114,115,116,117,118,119,120,121,122] 176,117 person-years [109] 3.99 million person-years [107,108]
			HR 1.12–21.16 [52,98,102,106,113,114,115,116,117,118,119] RR 1.44–3.74 [31,120] OR 1.19–3.29 [110,121,122]	Direction maintained HR 1.57–3.46 [102,106,115,116,118,119] OR 1.19–1.65 [121,122]	Western-type diet, unhealthy patterns (high-fat diet, family history, smoking, high BMI, antihypertensive meds, high inflammation, low physical activity, high n-3 PUFA intake)	

Abbreviations. UK = United Kingdom; HR = Hazard ratio; OR = Odds ratio; RR = Relative risk; IRR = Incidence rate ratio; BMI = Body Mass Index; IFG = Impaired Fasting Glucose; IGT = Impaired Glucose Tolerance; SFA = Saturated Fatty Acid; UPF = Ultra-Processed Food; ASB = Artificially Sweetened Beverage; PREDIMED = Prevención con Dieta Mediterránea; PNNS = Programme National Nutrition Santé; PUFA = Polyunsaturated Fatty Acid; ND = No Data (for factors that were not included in the high-quality studies).

## Data Availability

No new data were created or analyzed in this study. Data sharing is not applicable to this article.

## Supplementary Materials

The following supporting information can be downloaded at: https://www.mdpi.com/article/10.3390/nu17223507/s1, File S1: Customized search strategies utilized for every electronic database, alongside the respective count of studies retrieved from each database, Table S1: Quality assessment of included observational studies with the use of NHLBI quality assessment tool for cohort and cross-sectional studies [24], Table S2: Summary of studies investigating each food category and T2DM incidence, by country, region, author, exposure, follow-up time and effect estimate.
