# Supplementary material for: Nutritional Determinants of Type 2 Diabetes Mellitus in the European Union: A Systematic Review
Source: Nutrients. 2025 Nov 9;17(22):3507. doi: 10.3390/nu17223507 (PMC12655033; doi:10.3390/nu17223507)
Supplement: Supplementary file 1 [file nutrients-17-03507-s001.zip › File S1. Customized search strategies utilized for every electronic database, alongside the respective count of studies retrieved from each database.pdf]

Supplementary File S1. Customized search strategies utilized for every electronic database, alongside the respective count of studies retrieved from each database.

PubMed

(((("observational study"[Publication Type]) OR ("clinical trial"[Publication Type]) OR (clinicaltrial[Filter]) OR ("Clinical Studies as Topic"[Mesh]) OR (Controlled Before-After Studies[MeSH Terms]) OR (Case-Control Studies[MeSH Terms]) OR ("Cohort Studies"[Mesh])) AND (type 2 diabetes mellitus[MeSH Terms])) AND (("diet, food, and nutrition"[MeSH Terms] OR "Risk Factors"[MeSH Terms]) OR "Life Style"[MeSH Terms] OR "Exercise"[MeSH Terms] OR "Leisure Activities"[MeSH Terms] OR "Preventive Health Services"[MeSH Terms] OR "Communication"[MeSH Terms] OR "Health Behavior"[MeSH Terms] OR "Risk Reduction Behavior"[MeSH Terms] OR "Dietary Supplements"[MeSH Terms] OR "primary prevention"[MeSH Terms] OR "prevention and control"[MeSH Subheading] OR "Social Determinants of Health"[Mesh])) AND ((Austria[MeSH Terms] OR Belgium[MeSH Terms] OR Bulgaria[MeSH Terms] OR Croatia[MeSH Terms] OR Cyprus[MeSH Terms] OR Czech Republic[MeSH Terms] OR Denmark[MeSH Terms] OR Estonia[MeSH Terms] OR Finland[MeSH Terms] OR France[MeSH Terms] OR Germany[MeSH Terms] OR Greece[MeSH Terms] OR Hungary[MeSH Terms] OR Ireland[MeSH Terms] OR Italy[MeSH Terms] OR Latvia[MeSH Terms] OR Lithuania[MeSH Terms] OR Luxembourg[MeSH Terms] OR Malta[MeSH Terms] OR Netherlands[MeSH Terms] OR Poland[MeSH Terms] OR Portugal[MeSH Terms] OR Romania[MeSH Terms] OR Slovakia[MeSH Terms] OR Slovenia[MeSH Terms] OR Spain[MeSH Terms] OR Sweden[MeSH Terms] OR United Kingdom[MeSH Terms] OR Europe[MeSH Terms]) OR (Austria[tiab] OR Belgium[tiab] OR Bulgaria[tiab] OR Croatia[tiab] OR Cyprus[tiab] OR Czech Republic[tiab] OR Denmark[tiab] OR Estonia[tiab] OR Finland[tiab] OR France[tiab] OR Germany[tiab] OR Greece[tiab] OR Hungary[tiab] OR Ireland[tiab] OR Italy[tiab] OR Latvia[tiab] OR Lithuania[tiab] OR Luxembourg[tiab] OR Malta[tiab] OR Netherlands[tiab] OR Poland[tiab] OR Portugal[tiab] OR Romania[tiab] OR Slovakia[tiab] OR Slovenia[tiab] OR Spain[tiab] OR Sweden[tiab] OR United Kingdom[tiab] OR Europe[tiab] OR European Union[tiab]))

Hits: 3936

Scopus

(TITLE-ABS-KEY ((lifestyle AND modification*) OR (life AND style AND modification*) OR (life AND style AND change*) OR (lifestyle AND change*) OR (preventive AND health AND service*) OR (preventive AND service*) OR prophylaxis OR (disease AND prevention) OR (disease AND prophylaxis) OR (health AND protection) OR prevention OR (preventive AND medication) OR (preventive AND therapy) OR (preventive AND treatment) OR (prophylactic AND institution) OR (prophylactic AND management) OR (prophylactic AND medication) OR (prophylactic AND therapy) OR (prophylactic AND treatment) OR prophylaxis OR exercise OR effort OR (exercise AND capacity) OR (exercise AND performance) OR (exercise AND training) OR exertion OR (fitness AND training) OR (fitness AND workout) OR (physical AND conditioning) OR (physical AND effort) OR (physical AND exercise) OR (physical AND exertion) OR (work-out) OR t OR (leisure AND activit*) OR (diet* AND therapy) OR (diet* AND intervention*) OR (diet* AND treatment*) OR diet* OR (nutrition* AND therapy) OR (health AND education) OR (health AND fair*) OR (health AND science* AND education) OR (diet* AND assessment*) OR (diet* AND evaluation) OR (nutrition* AND assessment*) OR (nutrition* AND evaluation) OR (risk AND reduction) OR (risk AND reduction AND behavi*) OR (supplement*) OR (primary AND prevention) OR (risk AND factor*) OR (health AND determinant*))) AND (TITLE-ABS-KEY ((cohort AND stud*) OR (clinical AND trial*) OR (case-control) OR (before-after

AND stud*) OR (observational AND stud*))) AND (TITLE-ABS-KEY (austria OR belgium OR bulgaria OR croatia OR cyprus OR czech OR czechia OR denmark OR estonia OR finland OR france OR germany OR greece OR hungary OR ireland OR italy OR latvia OR lithuania OR luxembourg OR malta OR netherlands OR poland OR portugal OR romania OR slovakia OR slovenia OR spain OR sweden OR united AND kingdom OR uk OR european OR europe)) AND (TITLE-ABS-KEY ("diabetes" OR "diabetic" OR "type 2 dm" OR "type ii dm" OR "dmt2" OR "t2 dm")) Embase ('clinical study'/exp OR 'observational study'/exp OR 'controlled study'/exp OR 'experimentalstudy'/exp OR 'prevention study'/exp OR 'field study'/exp) AND ('nutrition'/exp OR 'risk factor'/exp OR 'lifestyle'/exp OR 'physical activity, capacity andperformance'/exp OR 'leisure'/exp OR 'preventive health service'/exp OR 'mass communication'/expOR 'health behavior'/exp OR 'risk reduction'/exp OR 'dietary

supplement'/exp OR 'prevention andcontrol'/exp OR 'health determinants'/exp) AND ('austria'/exp OR 'belgium'/exp OR 'bulgaria'/exp OR 'croatia'/exp OR 'cyprus'/exp OR 'czechrepublic'/exp OR 'denmark'/exp OR 'estonia'/exp OR 'finland'/exp OR 'france'/exp OR 'germany'/exp OR'greece'/exp OR 'hungary'/exp OR 'ireland'/exp OR 'italy'/exp OR 'latvia'/exp OR 'lithuania'/exp OR'luxembourg'/exp OR 'malta'/exp OR 'netherlands'/exp OR 'poland'/exp OR 'portugal'/exp OR'romania'/exp OR 'slovakia'/exp OR 'slovenia'/exp OR 'spain'/exp OR 'sweden'/exp OR 'unitedkingdom'/exp OR 'european union'/exp OR 'europe'/exp) AND 'austria'/exp OR 'belgium'/exp OR 'bulgaria'/exp OR 'croatia'/exp OR 'cyprus'/exp OR 'czechrepublic'/exp OR 'denmark'/exp OR 'estonia'/exp OR 'finland'/exp OR 'france'/exp OR 'germany'/exp OR'greece'/exp OR 'hungary'/exp OR 'ireland'/exp OR 'italy'/exp OR 'latvia'/exp OR 'lithuania'/exp OR'luxembourg'/exp OR 'malta'/exp OR 'netherlands'/exp OR 'poland'/exp OR 'portugal'/exp OR'romania'/exp OR 'slovakia'/exp OR 'slovenia'/exp OR 'spain'/exp OR 'sweden'/exp OR 'unitedkingdom'/exp OR 'european union'/exp OR 'europe'/exp) AND ('non insulin dependent diabetes mellitus'/exp)

Hits: 6076

CINHAL Plus

((MH "NonexperimentalStudies+") OR (MH"Experimental Studies+")) AND ((MH "Diet+") OR (MH"Exercise+") OR (MH"Leisure Activities+") OR(MH "Preventive HealthCare+") OR (MH "HealthBehavior+") OR (MH"Communication+") OR(MH "Primary HealthCare") OR (MH "DietarySupplements+") OR (MH"Risk Taking Behavior+")) AND ((MM "Diabetes Mellitus,Type 2")) limited to Europe

Hits: 2181

Web of Science

TS=(insulin independent diabetes OR insulin independent diabetes mellitus OR maturity onset diabetes OR maturity onset diabetes mellitus OR maturity onset diabetes of the young OR non insulin dependent diabetes OR non-insulindependent diabetes mellitus OR noninsulin dependent diabetes OR type 2 diabetes OR type 2 diabetes mellitus OR type ii

diabetes OR type ii diabetes mellitus OR non insulin dependent diabetes mellitus OR niddm* OR t2dm* OR adult onset diabetes OR diabetes mellitus type 2 OR diabetes mellitus type ii OR diabetes mellitus, maturity onset OR diabetes mellitus, non insulin dependent OR diabetes mellitus, non-insulin-dependent OR diabetes mellitus, type 2 OR diabetes mellitus, type ii OR diabetes type 2 OR diabetes type ii OR diabetes, adult onset OR dm) AND TS=(Austria OR Belgium OR Bulgaria OR Croatia OR Cyprus OR Czech Republic OR Czechia OR Denmark OR Estonia OR Finland OR France OR Germany OR Greece OR Hungary OR Ireland OR Italy OR Latvia OR Lithuania OR Luxembourg OR Malta OR Netherlands OR Poland OR Portugal OR Romania OR Slovakia OR Slovenia OR Spain OR Sweden OR United Kingdom OR UK OR Europe*) AND TS=(lifestyle modification* OR life style modification* OR life style change* OR lifestyle change* OR preventive health service* OR preventive service* OR prophylaxis OR disease prevention OR disease prophylaxis OR health protection OR prevention, disease OR preventive medication OR preventive therapy OR preventive treatment OR prophylactic institution OR prophylactic management OR prophylactic medication OR prophylactic therapy OR prophylactic treatment OR prophylaxis OR exercise OR effort OR exercise capacity OR exercise performance OR exercise training OR exertion OR fitness training OR fitness workout OR physical conditioning OR physical effort OR physical exercise OR physical exertion OR physical work-out OR physical workout OR Leisure Activit* OR diet* therapy OR diet* intervention* OR diet* treatment* OR nutrition* therapy OR health education OR education, health OR health fair* OR health science* education OR diet* assessment* OR diet* evaluation OR nutrition* assessment* OR nutrition* evaluation OR risk reduction OR risk reduction behavi* OR supplement* OR primary prevention OR risk factor* OR health determinant*) AND TS=(randomized controlled trial* OR controlled trial*, randomized OR randomised controlled stud* OR randomised controlled trial* OR randomized controlled stud* OR trial*, randomized controlled OR cohort analysis OR analysis, cohort OR cohort life cycle OR cohort stud* OR case control stud* OR case-control stud* OR control study, case OR matched case control OR matched case control stud* OR matched case-control stud* OR observation* stud* OR non experimental stud* OR nonexperimental stud*)

Hits: 4687

CAB abstracts

("diet" OR "dietary factors" OR "nutrition" OR "food" OR "risk factors" OR "risk reduction" OR "risk management" OR "risk groups" OR "lifestyle" OR "lifestyles" OR "exercise" OR "leisure activities" OR "leisure behaviour" OR "counselling" OR "preventive medicine" OR "communication" OR "health behaviour" OR "health determinants" OR "health education" OR "supplements" OR "prevention") AND (("type 2 diabetes" OR "noninsulin-dependent diabetes mellitus" OR "NIDDM" OR "diabetes mellitus type 2") AND ("cohort studies" OR "case control studies" OR "clinical trials" OR "observational studies") AND (austria OR belgium OR bulgaria OR croatia OR cyprus OR czech OR czechia OR denmark OR estonia OR finland OR france OR germany OR greece OR hungary OR ireland OR italy OR latvia OR lithuania OR luxembourg OR malta OR netherlands OR poland OR portugal OR romania OR slovakia OR slovenia OR spain OR sweden OR united AND kingdom OR uk OR european OR europe))

Hits: 1598

Clinicaltrials.gov

Hits: 2
