# Supplementary material for: Nutritional Determinants of Type 2 Diabetes Mellitus in the European Union: A Systematic Review
Source: Nutrients. 2025 Nov 9;17(22):3507. doi: 10.3390/nu17223507 (PMC12655033; doi:10.3390/nu17223507)
Supplement: Supplementary file 1 [file nutrients-17-03507-s001.zip › Table S1. Quality assessment.pdf]

Table S1. Quality assessment of included observational studies with the use of NHLBI quality assessment tool for cohort and cross-sectional studies [24].

References	Q1	Q2	Q3	Q4	Q5	Q6	Q7	Q8	Q9	Q10	Q11	Quality
Becerra-Tomas et al. (2018) [10]	Y	Y	Y	Y	Y	Y	Y	Y	N	Unclear	Y	L
Conklin et al. (2016) [25]	Y	Y	Unclear	Y	Y	Y	Y	Y	Y	NA	Y	H
Andre et al. (2020) [26]	Y	Y	Y	Y	Y	Y	Y	Y	Y	Unclear	Y	H
Ruiz-Estigarribia et al. (2020) [27]	Y	Y	Y	Y	Y	Y	Y	Y	Y	Y	Y	H
Filippatos et al. (2016) [28]	Y	Y	Y	Y	Y	Y	Y	Y	Y	Unclear	Y	H
Heidemann et al. (2005) [29]	Y	Y	Y	Y	Y	Y	Y	N	Y	NA	Y	H
Chen et al. (2018) [30]	Y	Y	Y	Y	Y	Y	Y	Y	N	Unclear	Y	L
Montonen et al. (2005) [31]	Y	Y	Y	Y	Y	Y	Y	Y	Unclear	Unclear	Y	M
Mursu et al. (2014) [32]	Y	Y	Y	Y	Y	Y	Y	Y	Y	NA	Y	H
Montonen et al. (2003) [33]	Y	Y	Y	Y	Y	Y	Y	Y	Y	N	Y	H
Soriguer et al. (2013) [34]	Y	Y	Y	Y	Y	Y	Y	Y	N	Unclear	Y	L
Cooper et al. (2012) [35]	Y	Y	Y	Y	Y	Y	Y	Y	Unclear	Unclear	Y	M
Kosti et al. (2023) [36]	Y	Y	Y	Y	Y	Y	Y	Y	Y	Y	Y	H
Ibsen et al. (2021) [37]	Y	Y	Y	Y	Y	Y	Y	Y	Y	Y	Y	H
Romaguera et al. (2013) [38]	Y	Y	Y	Y	Y	Y	Y	Y	N	Y	Y	L
Ahmadi-Abhari et al. (2014) [39]	Y	Y	Y	Y	Y	Y	Y	Y	Y	Y	Y	H
Díaz-López et al. (2016) [40]	Y	Y	Y	Y	Y	Y	Y	Y	Unclear	Y	Y	M
Johansson et al. (2019) [41]	Y	Y	Y	Y	Y	Y	Y	Y	Y	N	Y	H
Lajous et al. (2015) [42]	Y	Y	Y	Y	Y	Y	N	Y	Y	Y	Y	H

References	Q1	Q2	Q3	Q4	Q5	Q6	Q7	Q8	Q9	Q10	Q11	Quality
Männistö et al. (2010) [43]	Y	Y	Y	Y	Y	Y	Y	Y	Y	Y	Y	H
Lajous et al. (2012) [44]	Y	Y	Y	Y	Y	Y	N	Y	Y	Y	Y	H
Van Nielen et al. (2014) [45]	Y	Y	Y	Y	Y	Y	Y	Y	N	N	Y	L
Fagherazzi et al. (2017) [46]	Y	Y	Y	N	Y	Y	Y	Y	Y	N	Y	H
Levy et al. (2021) [47]	Y	Y	Unclear	Y	Y	Y	Y	Y	Y	Y	Y	H
Löfvenborg et al. (2016) [48]	Y	Y	Y	Y	Y	Y	Unclear	Y	N	Y	Y	L
O'Connor et al. (2015) [49]	Y	Y	Y	Y	Y	Y	Y	Y	Unclear	Unclear	Y	M
Wallin et al. (2017) [50]	Y	Y	Y	Y	Y	Y	Y	Y	N	Unclear	Y	L
Van woudenbergh et al. (2009) [51]	Y	Y	Y	Y	Y	Y	Y	Y	N	NA	Y	L
Rajaobelina et al. (2019) [52]	Y	Y	Y	Y	Y	Y	Y	Y	N	N	Y	L
Chen et al. (2021) [53]	Y	Y	Y	Y	Y	Y	Y	Y	Unclear	Unclear	Y	M
Barouti et al. (2022) [54]	Y	Y	Y	Y	Y	Y	Y	Y	Unclear	Unclear	Y	M
Stuber et al. (2021) [55]	Y	Y	Y	Y	Y	Y	Y	Y	Y	NA	Y	H
Scheffers et al. (2020) [56]	Y	Y	Y	Y	Y	Y	Y	Y	N	Y	Y	L
Ibsen et al. (2017) [57]	Y	Y	Y	Y	Y	Y	Y	Y	Y	NA	Y	H
Sluijs et al. (2012) [58]	Y	Y	Y	Y	Y	Y	Y	Y	Y	Unclear	Y	H
Cooper et al. (2012) [59]	Y	Y	Y	Y	Y	Y	Unclear	Y	Unclear	Unclear	Y	M
Zazpe et al. (2013) [60]	Y	Y	Y	Y	Y	Y	Y	Y	Y	N	Y	H
Patel et al. (2009) [61]	Y	Y	Y	Y	Y	Y	Y	Y	N	Unclear	Y	L

References	Q1	Q2	Q3	Q4	Q5	Q6	Q7	Q8	Q9	Q10	Q11	Quality
Bergholdt et al. (2015) [62]	Y	Y	Y	Y	Y	Y	Y	Y	Unclear	Unclear	Y	M
Noerman et al. (2019) [63]	Y	Y	Unclear	Y	Y	Y	Y	Y	Unclear	N	Y	M
Lecomte et al. (2007) [64]	Y	Y	Y	Y	Y	Unclear	Y	Y	Unclear	Unclear	Y	L
Ahmed et al. (2020) [65]	Y	Y	Y	Y	Y	Y	Y	Y	Y	NA	Y	H
Radzevičienė et al. (2012) [66]	Y	Y	Y	Y	Y	Y	Y	Unclear	Unclear	Unclear	Y	M
Mamluk et al. (2017) [67]	Y	Y	Y	Y	Y	Y	Unclear	Y	Unclear	Unclear	Y	M
Pertiwi et al. (2020) [68]	Y	Y	Y	Y	Y	Y	Y	Y	Y	NA	Y	H
Virtanen et al. (2017) [69]	Y	Y	Y	Y	Y	Y	Y	Y	N	Unclear	Y	M
Krachler et al. (2008) [70]	Y	Y	Y	Y	Y	Y	Y	Y	N	Unclear	Y	L
Ibsen et al. (2020) [71]	Y	Y	Y	Y	Y	Y	Y	Y	N	N	Y	L
Van woudenbergh et al. (2012) [72]	Y	Y	Y	Y	Y	Y	Y	Y	Y	Unclear	Y	H
Sluijs et al. (2010) [73]	Y	Y	Y	Y	Y	Y	Y	Y	N	N	Y	M
Chen et al. (2020) [74]	Y	Y	Y	Y	Y	Y	Y	Y	Unclear	N	Y	M
The InterAct Consortium (2013) [75]	Y	Y	Y	Y	Y	Y	Y	Y	N	N	Y	L
Olsson et al. (2021) [76]	Y	Y	Y	Y	Y	Y	Y	Y	N	N	Y	L
Llavero-Valero et al. (2021) [77]	Y	Y	Y	Y	Y	Y	Y	Y	Y	Y	Y	H
Srour et al. (2020) [78]	Y	Y	Y	Y	Y	Y	Y	Y	Y	Unclear	Y	H
Ma et al. (2020) [79]	Y	Y	Y	Y	Y	Y	Y	Y	N	Y	Y	L
Sartorelli et al. (2010) [80]	Y	Y	Y	Y	Y	Y	Y	Y	N	Y	Y	L
Fresan et al. (2017) [81]	Y	Y	Y	Y	Y	Y	Y	Y	N	Y	Y	L

References	Q1	Q2	Q3	Q4	Q5	Q6	Q7	Q8	Q9	Q10	Q11	Quality
Hu et al. (2006) [82]	Y	Y	Y	Y	Y	Y	Y	Y	N	Y	Y	L
Tuomilehto et al. (2004) [83]	Y	Y	Y	Y	Y	Y	Y	Y	N	Y	Y	L
Bidel et al. (2008) [84]	Y	Y	Y	Y	Y	Y	Y	N	Y	NA	Y	H
Hamer et al. (2008) [85]	Y	Y	Y	Y	Y	Y	Y	Y	N	N	Y	L
Said et al. (2020) [86]	Y	Y	Y	Y	Y	Y	Y	Y	N	N	Y	L
Van Dieren et al. (2009) [87]	Y	Y	Y	Y	Y	Y	N	Y	N	N	Y	L
Imamura et al. (2019) [88]	Y	Y	Y	Y	Y	Y	Y	N	N	N	Y	L
The InterAct Consortium (2012) [89]	Y	Y	Y	Y	Y	Y	Y	Y	N	N	Y	L
Fagherazzi et al. (2013) [90]	Y	Y	Y	Y	Y	Y	Y	Y	Y	Y	Y	H
Floegel et al. (2012) [91]	Y	Y	Y	Y	Y	Y	Y	Y	Y	Y	Y	H
Montonen et al. (2007) [92]	Y	Y	Y	Y	Y	Y	Y	Y	Y	N	Y	H
Sluijs et al. (2013) [93]	Y	Y	Y	Y	Y	Y	Y	Y	Unclear	N	Y	M
Sluijs et al. (2010) [94]	Y	Y	Y	Y	Y	Y	Y	Y	N	Unclear	Y	L
Boonpor et al. (2022) [95]	Y	Y	Y	Y	Y	Y	Y	Y	Y	NA	Y	H
Cea-Soriano et al. (2022) [96]	Y	Y	Y	Y	Y	Y	Y	Y	Uncear	Y	Y	M
Rossi et al. (2013) [97]	Y	Y	Y	Y	Y	Y	Y	Y	N	NA	Y	L
Iqbal et al. (2019) [98]	Y	Y	Y	Y	Y	Y	Y	Y	N	Unclear	Y	L
Mandalazi et al. (2016) [99]	Y	Y	Y	Y	Y	Y	Y	Y	Y	Y	Y	H
Kesse-Guyot et al. (2020) [100]	Y	Y	Y	Y	Y	Y	Y	N	N	Unclear	Y	L

References	Q1	Q2	Q3	Q4	Q5	Q6	Q7	Q8	Q9	Q10	Q11	Quality
Kesse-Guyot et al. (2021) [101]	Y	Y	Y	Y	Y	Y	Y	Y	Uclear	Y	Y	M
Markanti et al. (2021) [102]	Y	Y	Y	Y	Y	Y	Y	Y	Y	Unclear	Y	H
Lacoppidan et al. (2015) [103]	Y	Y	Y	Y	Y	Y	Y	Y	N	N	Y	L
Mozaffarian et al. (2007) [104]	Y	Y	Y	Y	Y	Y	Y	Y	Unclear	Y	Y	M
The InterAct Consortium (2014) [105]	N	Y	Y	Y	Y	Y	Y	Y	N	N	Y	L
Buijsse et al. (2015) [106]	Y	Y	Y	Y	Y	Y	Y	Y	Y	N	Y	H
Patel et al. (2012) [107]	Y	Y	Unclear	Y	Y	Y	Y	Y	Unclear	Unclear	Y	M
The InterAct Consortium (2013) [108]	Y	Y	Y	Y	Y	Y	Y	Y	N	Y	Y	L
Schulze et al. (2008) [109]	Y	Y	Y	Y	Y	Y	Y	Unclear	Y	Y	Y	H
Simmons et al. (2007) [110]	Y	Y	Y	Y	N	Y	Y	Y	N	Y	Y	L
Herzog et al. (2021) [111]	Y	Y	Y	Y	Y	NA	Y	Unclear	Y	NA	Y	H
Martınez-Gonzalez et al. (2008) [112]	Y	Y	Y	Y	Y	Y	Y	Y	Unclear	Y	Y	M
GaoMin et al. (2022) [113]	Y	Y	Y	Y	Y	Y	Y	Y	N	Y	Y	L
Eguaras et al. (2017) [114]	Y	Y	Y	Y	Y	Y	Y	Y	Unclear	Unclear	Y	M
Vassou et al. (2021) [115]	Y	Y	Y	Y	Y	Y	Y	Y	Y	N	Y	H
Kouvari et al. (2022) [116]	Y	Y	Y	Y	Y	Y	Y	Y	Y	NA	Y	H
Eckel et al. (2015) [117]	Y	Y	Y	Y	Y	Y	Y	Y	Unclear	N	Y	M
Dow et al. (2016) [118]	Y	Y	Y	Y	Y	Y	Y	Y	Y	Y	Y	H
Tertsunen et al. (2021) [119]	Y	Y	Y	Y	Y	Y	Y	Y	Y	NA	Y	H

References	Q1	Q2	Q3	Q4	Q5	Q6	Q7	Q8	Q9	Q10	Q11	Quality
Long et al. (2015) [120]	Y	Y	Y	Y	Y	Y	Y	Y	Unclear	Unclear	Y	M
Brayner et al. (2021) [121]	Y	Y	Y	Y	Y	Y	Y	Y	Y	NA	Y	H
MingJie et al. (2021) [122]	Y	Y	Y	Y	Y	Y	Y	N	Y	Y	Y	H
Cejudo et al. (2021) [126]	Y	Y	Y	Y	Y	Y	Y	Y	Y	Y	Y	H
Brouwer-Brolsma et al. (2016) [127]	Y	Y	Y	Y	Y	Y	Y	Y	Y	N	Y	H
Struijk et al. (2013) [128]	Y	Y	Y	Y	Y	Y	Y	Y	Unclear	Unclear	Y	M
Marí-Sanchis et al. (2011) [131]	Y	Y	Y	Y	Y	Y	Y	Y	Y	Y	Y	H
Soedamah-Muthu et al. (2013) [133]	Y	Y	Y	Y	Y	Y	Y	Y	Y	Unclear	Y	H

[a] Y: Yes; N: No; CD: Cannot determine; NA: Not applicable; NR: Not reported

[b] Assessment questions:

Q1: Were the two groups similar and recruited from the same population?

Q2: Were the exposures measured similarly to assign people to both exposed and unexposed groups?

Q3: Was the exposure measured in a valid and reliable way?

Q4: Were confounding factors identified?

Q5: Were strategies to deal with confounding factors stated?

Q6: Were the groups/participants free of the outcome at the start of the study (or at the moment of exposure)?

Q7: Were the outcomes measured in a valid and reliable way?

Q8: Was the follow up time reported and sufficient to be long enough for outcomes to occur?

Q9: Was follow up complete, and if not, were the reasons to loss to follow up described and explored?

Q10: Were strategies to address incomplete follow up utilized?

Q11: Was appropriate statistical analysis used?
