# Supplementary material for: Nutritional Determinants of Type 2 Diabetes Mellitus in the European Union: A Systematic Review
Source: Nutrients. 2025 Nov 9;17(22):3507. doi: 10.3390/nu17223507 (PMC12655033; doi:10.3390/nu17223507)
Supplement: Supplementary file 1 [file nutrients-17-03507-s001.zip › Table S2. Summary of studies.pdf]

Table S2. Summary of studies investigating each food category and T2DM incidence, by country, region, author, exposure, follow-up time and effect estimate.

Country	Region	Author, Year	Exposure Summary	Follow-up time	Effect Estimate (95% CI)
			Whole & Minimally Processed Foods		
Finland	Kuopio, Eastern Finland	Mursu, 2014 [32]	High berry intake (men)	19.3 years	HR = 0.65 (95% CI: 0.49–0.88)
	Various regions	Montonen, 2003 [33]	Whole grain intake 163-237 g/day	10 years	RR = 0.49 (95% CI: 0.29–0.82)
	Kuopio, Eastern Finland	Noerman, 2019 [63]	Metabolomic profile linked to egg intake (men)	19.3 years	OR = 1.94, 95% CI: 1.45–2.60 (tyrosine), OR = 2.13, 95% CI: 1.57–2.88 (hexose-containing compound).
France	Not available	Lajous, 2015 [42]	Egg consumption (5 or more per week); High dietary cholesterol (≥454 mg/day) (women)	13.8 years	HR = 1.00 (95% CI: 0.78–1.29); HR = 1.40 (95% CI: 1.19–1.63)
	Not available	Lecomte, 2007 [64]	No daily dairy consumption (predictive in IFG men)	5 years ± 6 months	OR = 1.7 (95% CI: 1.2–2.6)
Spain	Not available	Becerra-Tomas, 2018 [10]	Legume consumption (lentils, chickpeas) (men and women, mainly women)	4.3 years	Total legume intake (HR = 0.65, 95% CI: 0.43–0.96), lentils (HR = 0.69, 95% CI: 0.50–0.96; HR = 0.66, 95% CI: 0.46–0.94; HR = 0.67, 95% CI: 0.46–0.98), chickpeas (HR = 0.68, 95% CI: 0.46–1.00)
	Southern Spain, Pizarra	Soriguer, 2013 [34]	White rice intake and metabolic risk factors (men and women, mainly women)	6 years	OR = 0.41, 95% CI: 0.17–0.98 (white rice), OR = 3.31, 95% CI: 1.52–7.22 (BMI ≥30), OR = 5.08, 95% CI: 2.01–12.79 (IFG), OR = 8.07, 95% CI: 3.79–19.96 (IGT), OR = 1.03, 95% CI: 1.01–1.06 (age).

Country	Region	Author, Year	Exposure Summary	Follow-up time	Effect Estimate (95% CI)
	Southeastern, Spain, Navarra	Zazpe, 2013 [60]	Egg consumption >4 eggs/week (men and women, mainly women)	6 years	OR = 0.70 (95% CI: 0.30–1.70)
	Not available	Díaz-López, 2016 [40]	High yogurt intake (men and women, mainly women)	4.1 years	HR = 0.70 (95% CI: 0.51–0.95)
UK	England, Wales, Scotland	Chen, 2021 [53]	Oily fish intake ≥2 servings/week (men and women, mainly women)	10.1 years	HR = 0.78 (95% CI: 0.71–0.86); per serving HR = 0.92 (95% CI: 0.89–0.96)
	Norwich	Patel, 2009 [61]	Intake of total fish and specific fish types (men and women, mainly women)	10.2 years	Total fish (OR = 0.75, 95% CI: 0.58–0.96; OR = 0.77, 95% CI: 0.61–0.96),<br>white fish (OR = 0.80, 95% CI: 0.69–0.94), and oily fish (OR = 0.83, 95% CI: 0.70–0.97);<br>shellfish intake (≥1 portion/week) (OR = 1.36, 95% CI: 1.02–1.81; OR = 1.53, 95% CI: 1.20–1.96)
	Southeast UK, London	Soedamah-Muthu, 2013 [133]	Dairy intake (men and women, mainly women)	10 years	Neutral associations
	Northeast, Norwich	Cooper, 2012 [35]	Fruit and vegetable variety intake (men and women, mainly women)	10.9 years	HR = 0.61 (95% CI: 0.48–0.78)
Sweden	Stockholm County	Ahmed, 2020 [65]	Low vegetable intake <2 servings/day (men)	4 years	OR = 2.08 (95% CI: 1.29–3.36)

Country	Region	Author, Year	Exposure Summary	Follow-up time	Effect Estimate (95% CI)
	Västerbotten and Norrbotten, Northern Sweden	Johansson, 2019 [41]	Non-fermented milk, fermented milk, cheese, and butter intake (men and women, mainly women)	14.2 years	High non-fermented milk intake (HR = 1.15, 95% CI: 1.05–1.26), moderate-to-high cheese intake (HR = 1.26, 95% CI: 1.14–1.40; HR = 1.19, 95% CI: 1.06–1.33), high fermented milk intake (HR = 0.85, 95% CI: 0.78–0.93; HR = 0.82, 95% CI: 0.75–0.91), and moderate butter intake (HR = 0.83, 95% CI: 0.76–0.92; HR = 0.88, 95% CI: 0.80–0.97)
	Örebro and Västmanland, Central Sweden	Wallin, 2017 [50]	Fried fish; shellfish (men)	15 years	HR = 1.13 (95% CI: 1.00–1.28); HR = 1.19 (95% CI: 1.04–1.35)
	Stockholm	Barouti, 2022 [54]	Fruit intake	20 years	HR = 0.81 (95% CI: 0.66–0.99, men), HR = 0.62 (95% CI: 0.50–0.77, men), HR = 0.77 (95% CI: 0.61–0.90, men), HR = 0.76 (95% CI: 0.60–0.96, men), HR = 0.79 (95% CI: 0.64–0.90, women)
Lithuania	Kaunas	Radzevičienė, 2012 [66]	High egg consumption ≥3 eggs per week (both, mainly women)	Not available	OR = 3.02 (95% CI: 1.14–7.98)
	Ommoord, Rotterdam	Van Woudenbergh, 2009 [51]	Fish consumption (men and women, mainly women)	12 years	High total fish intake (RR = 1.32, 95% CI: 1.02–1.70), high lean fish intake (RR = 1.30, 95% CI: 1.01–1.68)
	Not available	Stuber, 2021 [55]	Dairy substitutions (women)	15.3 years	HR = 0.93, 95% CI: 0.60–1.44; HR = 1.07, 95% CI: 0.89–1.29)
Netherlands	Leyenburg, The Hague, The Netherlands	Cejudo, 2021 [126]	Dairy intake post-MI (men and women, mainly women)	3.3 years	No significant association
	Rotterdam, Ommoord	Brouwer-Brolsma, 2016 [127]	Total/specific dairy intake (men and women, mainly women)	9.5 years	No significant associations

Country	Region	Author, Year	Exposure Summary	Follow-up time	Effect Estimate (95% CI)
	Amsterdam, Doetinchem, Maastricht, Utrecht	Scheffers, 2020 [56]	Pure fruit juice and total fruit intake (women)	14.6 years	4 to <8 glasses/week HR = 0.92 (95% CI: 0.79–0.94)
Denmark	Eastern Denmark, Copenhagen	Struijk, 2013 [128]	Dairy intake (women, men)	5 years	No significant associations
	Copenhagen, Aarhus	Ibsen, 2017 [57]	Dairy Product Substitutions (men and women, mainly women)	15.3 years	Whole-fat yogurt vs. low-fat milk (HR = 0.72, 95% CI: 0.66–0.77), whole-fat yogurt vs. whole-fat milk (HR = 0.72, 95% CI: 0.66–0.78), whole-fat yogurt vs. buttermilk (HR = 0.74, 95% CI: 0.68–0.81), low-fat yogurt vs. whole-fat yogurt (HR = 1.40, 95% CI: 1.27–1.54), cheese vs. low-fat milk (HR = 0.97, 95% CI: 0.95–0.99), cheese vs. whole-fat milk (HR = 0.97, 95% CI: 0.96–0.99), cheese vs. low-fat yogurt (HR = 0.97, 95% CI: 0.95–0.99)
	Copenhagen	Bergholdt, 2015 [62]	Milk intake, lactase persistence genotype, and metabolic outcomes (men and women, mainly women)	5.5 years	Q2 (1–3 drinks/week) HR = 1.22, 95% CI: 1.04–1.44; Q5 (≥11 drinks/week) HR = 1.20, 95% CI: 1.03–1.40); Milk intake (HR = 1.10, 95% CI: 0.98–1.24), lactase persistence vs. nonpersistence (OR = 0.96, 95% CI: 0.86–1.08), milk consumers (OR = 0.88, 95% CI: 0.76–1.03), non-consumers (OR = 1.35, 95% CI: 1.07–1.70), 1 glass/week higher milk intake – genetic risk (OR = 0.99, 95% CI: 0.93–1.06).
Greece	Athens	Kosti, 2023 [36]	Specific vegetables (cruciferous, leafy, legumes)	10 years	All vegetables (HR = 0.29 (women), 95% CI: 0.16–0.53; HR = 0.56 (men), 95% CI: 0.34–0.92); allium vegetables (HR = 0.15 (women), 95% CI: 0.05–0.42); cruciferous vegetables (HR = 0.23 (women), 95% CI: 0.11–0.47); leafy vegetables (HR = 0.22 (women), 95% CI: 0.11–0.45); red/orange/yellow vegetables (HR = 0.29 (women), 95% CI: 0.16–0.52; HR = 0.54 (men), 95% CI: 0.33–0.89); legumes (HR = 0.27 (women), 95% CI: 0.15–0.50; HR = 0.57 (men), 95% CI: 0.35–0.92)
Multinational	Greece, Netherlands, Spain, Sweden	Mamluk, 2017 [67]	Leafy greens (men and women, mainly women)	1.8 years	Greece EPIC Elderly OR = 1.52 (95% CI: 1.13–2.04)

Country	Region	Author, Year	Exposure Summary	Follow-up time	Effect Estimate (95% CI)
	Denmark, France, Germany, Italy, Netherlands, Spain, Sweden, UK	Sluijs, 2012 [58]	Fermented dairy intake (men and women, mainly women)	12.3 years	Cheese intake (HR = 0.83, 95% CI: 0.70–0.98), combined fermented dairy (HR = 0.85, 95% CI: 0.73–0.99), yogurt and thick fermented milk (HR = 0.88, 95% CI: 0.80–0.98; HR = 0.82, 95% CI: 0.75–0.90), cheese (HR = 0.84, 95% CI: 0.74–0.95), combined fermented dairy (HR = 0.79, 95% CI: 0.72–0.87)
	France, Germany, The Netherlands, UK, Italy, Sweden, Denmark, Spain	Cooper, 2012 [59]	Fruit and vegetable intake (men and women, mainly women)	11 years	HR = 0.84 (95% CI: 0.73–0.96)
			Animal-based & Protein-Rich Foods		
Netherlands	Rotterdam, Ommoord	Van Woudenbergh, 2012 [72]	High processed meat intake Processed meat > 29.8 g/day (men and women, mainly women)	12.4 years	HR = 1.87 (95% CI: 1.26–2.78)
	Utrecht	Sluijs, 2010 [73]	Total, animal, and vegetable protein intake and macronutrient substitution (both, mainly women)	10 years	Total protein intake: HR = 1.50 (95% CI: 1.23–1.84), HR = 1.85 (95% CI: 1.53–2.25); Animal protein intake: HR = 1.35 (95% CI: 1.10–1.65), HR = 1.73 (95% CI: 1.43–2.10); Substituting total protein for fat: HR = 1.31 (95% CI: 1.06–1.61), for carbohydrates: HR = 1.28 (95% CI: 1.01–1.61).
	Rotterdam	Chen, 2020 [74]	Animal protein from meat, fish, and dairy (men and women, mainly women)	5.7–7.2 years	Meat (HR = 1.40, 95% CI: 1.12–1.75), fish (HR = 1.65, 95% CI: 1.30–2.10), and dairy (HR = 1.23, 95% CI: 1.00–1.49)
	Not available	Pertiwi, 2020 [68]	Plasma and dietary linoleic acid (mainly men)	3.4 years	HR = 0.81 (95% CI: 0.50–1.31)

Country	Region	Author, Year	Exposure Summary	Follow-up time	Effect Estimate (95% CI)
Finland	Southwestern	Männistö, 2010 [43]	Total and processed meat intake (men)	12 years	RR = 1.50 (95% CI: 1.19–1.89) and 1.37 (95% CI: 1.11–1.71)
	Eastern Finland, Kuopio	Virtanen, 2017 [69]	Plant protein intake (men)	19.3 years	HR = 0.71 (95% CI: 0.46–1.10)
Sweden	Northern Sweden, Umea	Krachler, 2008 [70]	Odd-chain saturated fatty acids (15:0, 17:0) (men and women, mainly women)	5.4 ± 2.6 years	HR = 0.71 (95% CI: 0.52–0.97) and 0.54 (95% CI: 0.35–0.83)
France	Not available	Lajous, 2012 [44]	Red and processed meat intake (Diet + unprocessed meat) (women)	13.8 years	HR = 1.30 (95% CI: 1.07–1.59)
Denmark	Aarhus and Copenhagen	Ibsen, 2021 [37]	Substitution of red meat with yogurt, eggs, grains (men and women, mainly women)	10 years	Eggs (−2.7; 95% CI: −4.0 to −1.4),<br>whole grains (−1.9; 95% CI: −2.7 to −1.1),<br>yogurt (−1.6; 95% CI: −2.5 to −0.8),<br>milk (−1.2; 95% CI: −2.1 to −0.4),<br>cheese (−1.0; 95% CI: −1.9 to −0.1),<br>poultry (−1.4; 95% CI: −3.2 to 0.3),<br>refined grains (−1.3; 95% CI: −2.2 to −0.5),<br>fish (−0.1; 95% CI: −1.8 to 1.6).

Country	Region	Author, Year	Exposure Summary	Follow-up time	Effect Estimate (95% CI)
Multinational	Denmark, France, Germany, Italy, the Netherlands, Spain, Sweden, UK	The InterAct Consortium, 2013 [75]	Total, red, and processed meat intake	11.7 years	Total meat: HR = 1.27 (95% CI: 1.13–1.42), HR = 1.97 (95% CI: 1.78–2.16), HR = 1.38 (95% CI: 1.15–1.65, men), HR = 1.25 (95% CI: 1.08–1.45, women);<br>Red meat: HR = 1.20 (95% CI: 1.07–1.35), HR = 1.57 (95% CI: 1.42–1.73), HR = 1.30 (95% CI: 1.09–1.56, men);<br>Processed meat: HR = 1.16 (95% CI: 1.04–1.31), HR = 1.61 (95% CI: 1.47–1.76), HR = 1.34 (95% CI: 1.14–1.57, men);<br>Red and processed meat: HR = 1.18 (95% CI: 1.04–1.33), HR = 1.84 (95% CI: 1.67–2.02), HR = 1.42 (95% CI: 1.19–1.69, men);<br>Offals: HR = 1.11 (95% CI: 1.04–1.19);<br>Poultry: HR = 1.13 (95% CI: 1.03–1.24), HR = 1.19 (95% CI: 1.02–1.38, women);<br>Meat iron: HR = 1.16 (95% CI: 1.04–1.30), HR = 1.67 (95% CI: 1.52–1.83), HR = 1.18 (95% CI: 1.01–1.38, men), HR = 1.25 (95% CI: 1.06–1.49, men).
Multinational	Denmark, France, Germany, Greece, Italy, the Netherlands, Norway, Spain, Sweden and UK	Van Nielen, 2014 [45]	Total and animal protein intake (men and women)	12 years	Higher total protein HR = 1.17 (1.00–1.38),<br>animal protein HR = 1.22 (1.06–1.40);<br>by BMI: <25 HR = 1.08 (1.00–1.17), 25–30 HR = 1.04 (1.00–1.07), >30 HR = 1.06 (1.00–1.11);<br>in women: BMI 25–30 HR = 1.07 (1.01–1.14), BMI >30 HR = 1.19 (1.09–1.32)
Multinational	France, Italy, Spain, UK, Netherlands, Germany, Sweden, and Denmark	Ibsen, 2020 [71]	Substitution of meat with yogurt, cheese, fish (men and women)	12.3 years	Yogurt (HR = 0.90, 95% CI: 0.86–0.95),<br>cheese (HR = 0.90, 95% CI: 0.83–0.97),<br>or fish (HR = 0.91, 95% CI: 0.84–1.00).
			Processed & Discretionary Foods		
Sweden	Southern Sweden, Malmö	Olsson, 2021 [76]	Disaccharide intake; sugar-sweetened beverages (SSB); added sugar (men)	18.4 years	Monosaccharides basic model: HR 0.75 (95%CI: 0.68-0.83)<br>Disaccharides basic model: HR 0.80 (95%CI: 0.74-0.87)<br>Trisaccharides basic model: HR 0.73 (95%CI: 0.67-0.81)<br>Polysaccharides basic model: HR 0.78 (95%CI: 0.70-0.87)

Country	Region	Author, Year	Exposure Summary	Follow-up time	Effect Estimate (95% CI)
Spain	Navarra	Llavero-Valero, 2021 [77]	Ultra-processed food (UPF) intake (highest vs lowest tertile) (both, mainly women)	12 years	HR = 1.65 (95% CI: 1.14–2.38)
	Not available	Marí-Sanchis, 2011 [131]	Olive oil consumption (men and women)	5.7 years	No significant associations
France	Not available	Fagherazzi, 2017 [46]	Frequent use of artificial sweeteners (always or almost always) (women)	18 years	HR = 2.17 (95% CI: 1.97–2.38)
	Not available	Srour, 2020 [78]	Dietary index of discretionary nutrients (replaces FSAm-NPS DI with dietary fat, sodium, sugar, fiber) (both, mainly women)	6 years	HR = 1.19 (95% CI: 1.09–1.30)
UK	Not available	Levy, 2021 [47]	Ultra-processed food intake (10% increase and highest quartile) (men and women, mainly women)	5.4 years	HR = 1.12 (95% CI: 1.04–1.20); HR = 1.44 (95% CI: 1.04–2.02)

Country	Region	Author, Year	Exposure Summary	Follow-up time	Effect Estimate (95% CI)
Composite & Special-purpose foods					
UK	Not available	Ma, 2020 [79]	Glucosamine supplement use	8.1 years	HR = 0.83, 95% CI: 0.78–0.89 (overall), HR = 0.81, 95% CI: 0.74–0.89 (women),<br>HR = 0.86, 95% CI: 0.78–0.94 (men), HR = 0.81, 95% CI: 0.70–0.94 (age <55),<br>HR = 0.86, 95% CI: 0.80–0.92 (age ≥55), HR = 0.83, 95% CI: 0.77–0.91 (BMI <30), HR = 0.84, 95% CI: 0.76–0.93 (BMI ≥30),<br>HR = 0.81, 95% CI: 0.75–0.87 (physically active), HR = 0.83, 95% CI: 0.74–0.92 (unhealthy diet),<br>HR = 0.84, 95% CI: 0.77–0.91 (healthy diet), HR = 0.82, 95% CI: 0.75–0.90 (never smokers),<br>HR = 0.86, 95% CI: 0.77–0.95 (past smokers), HR = 0.80, 95% CI: 0.65–0.98 (current smokers),<br>HR = 0.84, 95% CI: 0.77–0.91 (lifestyle score 2–3), HR = 0.82, 95% CI: 0.68–0.98 (score 4),<br>HR = 0.88, 95% CI: 0.80–0.98 (low deprivation), HR = 0.85, 95% CI: 0.78–0.93 (high deprivation)
Beverages					
France	Not available	Fagherazzi, 2013 [90]	SSB >359 mL/week; ASB >603 mL/week; Fruit juice 448–967 mL/week (women)	8.1 years	HR = 1.30; 1.68; 1.18 (95% CI: 1.02–1.66; 1.19–2.39; 1.01–1.38)
	Not available	Sartorelli, 2010 [80]	High tea and moderate coffee consumption (women)	11 years	HR = 0.67 and 0.73 (95% CI: 0.58–0.78 and 0.61–0.87)
Spain	Navarra	Fresan, 2017 [81]	Substitution: freshly made juice for bottle juice (both, mainly women)	10.2 years	HR = 0.65 (95% CI: 0.43–0.98)

Country	Region	Author, Year	Exposure Summary	Follow-up time	Effect Estimate (95% CI)
Sweden	Southern Sweden, Scania County	Löfvenborg, 2016 [48]	SSB intake: >2 servings/day vs. <1 serving/day (Model 3); Subgroup (Artificially sweetened beverages, Model 3, >2 servings/day)	Not available	OR = 3.17 (95% CI: 1.45–6.93); OR = 2.42 (95% CI: 1.19–4.92)
Germany	Potsdam, Heidelberg, and surrounding areas	Floegel, 2012 [91]	Decaffeinated Coffee: 2–<3 cups/day vs. <1 cup/day	8.9 years	HR = 1.51 (95% CI: 1.15–2.00)
Finland	Eastern Finnish provinces, the Turku Loimaa region in southwestern Finland, northern province of Oulu. North Karelia and Kuopio, Helsinki capital area.	Hu, 2006 [82]	Coffee 3–6 cups/day; Coffee ≥7 cups/day (vs. 0–2 cups/day, adults 35–74 y)	13.4 years	HR = 0.75 (95% CI: 0.63–0.89); 0.61 (95% CI: 0.49–0.76)
	Eastern and southern finland, North Karelia and Kuopio, and in the Turku-Loimaa region	Tuomilehto, 2004 [83]	Coffee consumption: 3–4; 5–6; 7–9; ≥10 cups/day vs. ≤2 cups/day (adults aged 35–64)	12 years	HR = 0.76 (95% CI: 0.57–1.01); 0.54 (95% CI: 0.40–0.73); 0.55 (95% CI: 0.37–0.81); 0.39 (95% CI: 0.24–0.64)
	Various regions	Montonen, 2007 [92]	Total Sugar Intake (in bevergaes): 115 g, 136 g, 171 g/day vs. 92 g/day	12 years	HR = 1.28 (95% CI: 0.82–1.95); 1.12 (95% CI: 0.71–1.77); 1.42 (95% CI: 0.90–2.24)

Country	Region	Author, Year	Exposure Summary	Follow-up time	Effect Estimate (95% CI)
	Kuopio, North Karelia, Turku-Loimaa, Helsinki, Oulu	Bidel, 2008 [84]	Coffee intake: ≥7 cups/day vs. 0–2 cups/day (men and women)	Not available	HR = 0.64 (95% CI: 0.51–0.80)
	London	Hamer, 2008 [85]	Coffee and tea consumption (men and women, mainly men)	11.7 years	Total coffee (>3) (HR: 0.80, CI: 0.54–1.18)
UK	Not available	Said, 2020 [86]	301–360 mg/day. Caffeine intake (coffee) (both, mainly women)	8.1 years	HR = 0.76 (95% CI: 0.67–0.86)
	Not available	O'connor, 2015 [49]	Intake of soft drinks per serving, sweetened tea/coffee, sweetened milk beverages, artificially sweetened beverages (ASB), and fruit juice (both, mainly women)	10.8 years	Soft drinks: HR = 1.14, 95% CI: 1.01–1.32 (per serving, Model 3); Sweetened tea/coffee: HR = 1.35, 95% CI: 1.12–1.63 (Tertile 2, Model 3); Sweetened milk: HR = 1.35, 95% CI: 1.10–1.67 (Tertile 3, Model 3); ASB: HR =1.67, 95% CI: 1.33–2.11 (Tertile 3, Model 2); Fruit juice: HR = 0.65, 95% CI: 0.53–0.81 (Tertile 1, Crude)
Netherlands	Amsterdam, Doetinchem and Maastricht	Van Dieren, 2009 [87]	Coffee and tea consumption (both, men and women)	10 years	Coffee consumption >6 cups/day HR: 0.84 (95% CI: 0.65–1.08). Tea consumption 5 cups/day: HR = 0.68, CI = (0.52–0.89)

Country	Region	Author, Year	Exposure Summary	Follow-up time	Effect Estimate (95% CI)
Multinational	Denmark; France; Germany; Italy; Netherlands; Spain; Sweden; UK	Imamura, 2019 [88]	Sugary drinks replacement with coffee or tea (both men and women)	Not available	Sugar-sweetened beverages HR = 1.18 (1.08–1.28), milk HR = 1.10 (1.02–1.19), coffee HR = 0.91 (0.89–0.94), tea HR = 0.93 (0.87–0.98); substitution of SSB: coffee HR = 0.79 (0.72–0.88), tea HR = 0.78 (0.72–0.85); replacing fruit juice with coffee HR = 0.89 (0.83–0.95); replacing milk with coffee HR = 0.85 (0.78–0.92), with tea HR = 0.82 (0.74–0.91).
		The InterAct Consortium, 2012 [89]	Tea consumption >0–<1; 1–<4; ≥4 cups/day (vs. 0 cups/day; adults, mean age = 51.7)	12.4 years	HR = 1.03 (95% CI: 0.91–1.16); 0.93 (95% CI: 0.81–1.05); 0.84 (95% CI: 0.71–1.00)
		Romaguera, 2013 [38]	Daily sugar-sweetened soft drink intake (overall)	6.9 years	HR: 1.22 (1.09, 1.38)
			Carbohydrates		
UK	Norfolk	Ahmadi-Abhari, 2014 [39]	High intake of carbohydrates, glucose, and fructose (both, mainly women)	6.3 years	HR = 0.85, 0.83, 0.82 (95% CI: 0.77–0.93, 0.75–0.90, 0.75–0.91)

Country	Region	Author, Year	Exposure Summary	Follow-up time	Effect Estimate (95% CI)
Netherlands	Utrecht	Sluijs, 2010 [94]	Glycemic load, carbohydrate, starch, and fiber intake (both, mainly women)	10.1 ± 1.9 years	HR = 1.27, 1.20, 1.23, 0.89 (95% CI: 1.11–1.44, 1.01–1.42, 1.07–1.42, 0.82–0.98)
Multinational	Sweden	Sluijs, 2013 [93]	GI/GL and digestible carbohydrate (both, mainly women)	12 years	Glycemic index overall HR = 1.05 (0.95–1.16), glycemic load HR = 1.07 (0.95–1.20), digestible carbohydrate HR = 0.97 (0.86–1.10), sugar HR = 0.96 (0.86–1.07), starch HR = 1.05 (0.94–1.18)
	France, Italy, Germany, UK	Sluijs, 2013 [93]	Glycemic index and starch intake (both, mainly women)	12 years	France: HR = 1.30 and 1.38 (95% CI: 0.73–2.33 and 0.77–2.47); Italy: HR = 1.29 and 1.29 (95% CI: 0.96–1.73 and 0.92–1.80); Germany: HR = 0.94 (95% CI: 0.66-1.34); UK: HR = 1.33 (95% CI: 0.88–2.02)
			Dietary Patterns		
UK	Norfolk	Conklin, 2016 [25]	Total diet diversity score of 5 (both, mainly women)	10 ± 1.5 years	HR = 0.70 (95% CI: 0.51–0.95)
	Northwest UK	André, 2020 [26]	Mediterranean diet (direct + indirect effects, total) (both, mainly women)	6.1 years	HR = 0.96 (95% CI: 0.93–0.99); HR = 0.90 (95% CI: 0.87–0.92); HR = 0.86 (95% CI: 0.82–0.90)
	England, Wales, and Scotland	Gao, 2022 [113]	Unhealthy dietary patterns (highest quintile) (men and women)	8.4 years	HR = 1.38 (95% CI: 1.27–1.49)
		Boonpor, 2022 [95]	Fish eater's vs meat eaters (both, mainly women)	5.4 years	HR = 0.41 (95% CI: 0.31–0.55)

Country	Region	Author, Year	Exposure Summary	Follow-up time	Effect Estimate (95% CI)
		Brayner, 2021 [121]	High-fat dietary patterns (both, mainly women)	6.3 years	For overall obesity, DP2 Tertile 3 OR = 1.24 (1.02–1.50); for abdominal obesity, DP2 Tertile 3 OR = 1.19 (1.02–1.38)
	Eastern part of England (Norfolk region)	Simmons, 2007 [110]	Lifestyle + diet factors (men and women)	4.6 years	Family history (OR = 1.78, 95% CI: 1.20–2.60), smoking (OR = 1.53, CI: 1.51–2.03), BMI (OR = 2.08, CI: 1.80–2.40), antihypertensive meds (OR = 2.13, CI: 1.61–2.90); low activity (OR = 0.42, CI: 0.31–0.58 and OR = 0.72, CI: 0.64–0.82); low green leafy veg/veg/fruit/whole grain (OR = 0.60, CI: 0.40-0.90; 0.66, CI: 0.46-0.94; 0.69, CI: 0.52-0.91;0.72, CI: 0.53–0.97); low meat intake (OR = 3.29, CI: 1.02–10.61).
Spain	Navarra	Martínez-Gonzalez, 2008 [112]	Adherence to PREDIMED dietary pattern (high adherence) (both, mainly women)	4.4 years	IRR = 0.17 (95% CI: 0.04–0.72)
	Not available	Cea-Soriano, 2022 [96]	Mediterranean diet (multiple models) (men and women)	4.2 years	HR = 0.59 (95% CI: 0.42–0.84); HR = 0.63 (95% CI: 0.42–0.93); HR = 0.48 (95% CI: 0.27–0.86)
	Northern, Navarra	Ruiz-Estigarribia, 2020 [27]	Healthy lifestyle score (HLS 7–9) and Mediterranean diet adherence (men and women)	12 years	HR = 0.54 (95% CI: 0.30–0.99); HR = 0.70 (95% CI: 0.50–0.99)
	Navarra	Eguaras, 2017 [114]	BMI and Mediterranean diet adherence interaction (both, mainly women)	9.5 years	High BMI/low Mediterranean diet HR = 21.16 (95% CI: 7.82–57.26); Higher Mediterranean adherence HR = 14.76 (95% CI: 7.21–30.22)

Country	Region	Author, Year	Exposure Summary	Follow-up time	Effect Estimate (95% CI)
Greece	Not available	Rossi, 2013 [97]	Mediterranean diet score ≥6 (men and women)	11.34 years	HR = 0.88 (95% CI: 0.78–0.99)
	Athens	Vassou, 2021 [115]	Low Mediterranean diet adherence + high inflammation (both, mainly man)	10 years	HR = 3.46 (95% CI: 1.92–6.25)
		Filippatos, 2016 [28]	Mediterranean diet Score >35 (men and women)	10 years	OR = 0.12 (95% CI: 0.03–0.54)
		Kouvari, 2022 [116]	Plant-based diet indices (PDI) (men and women)	8.41 years	Total sample HR = 2.95 (2.01–4.23);<br>overall PDI <58 HR = 3.05 (1.96–4.72), ≥58 HR = 1.87 (1.09–3.41);<br>healthful PDI <48 HR = 3.12 (1.87–4.23), ≥48 HR = 1.79 (1.02–3.61);<br>unhealthful PDI <58 HR = 2.04 (1.19–3.76).
Germany	Not available	Heidemann, 2005 [29]	Healthy dietary scores (both, mainly men)	Not available	OR = 0.26 (95% CI: 0.12–0.56)
	Potsdam	Schulze, 2008 [109]	Intake of starch and sugars (sucrose, glucose, fructose) (men and women)	176,117 person-years	Starch (men): RR = 0.79 (95% CI: 0.50–1.24) – Q5;<br>Sucrose (men): RR = 0.72 (95% CI: 0.50–1.04) – Q5;<br>Glucose (women): RR = 0.78 (95% CI: 0.52–1.15) – Q2;<br>Fructose (women): RR = 0.75 (95% CI: 0.52–1.58) – Q5
		Eckel, 2015 [117]	Risk factors by BMI (men and women)	7 years	BMI <25 HR = 4.03 (95% CI: 1.32–12.3);<br>BMI ≥30 HR = 2.76 (95% CI: 1.55–4.91)

Country	Region	Author, Year	Exposure Summary	Follow-up time	Effect Estimate (95% CI)
	Not available	Iqbal, 2019 [98]	Dietary patterns and biomarkers (men and women)	15 years	Western-type diet: HR = 1.57 (95% CI: 1.14–2.16, women); High-fat dairy pattern: HR = 0.69 (95% CI: 0.54–0.89, men), HR = 0.71 (95% CI: 0.53–0.96, women). Biomarkers (men): LDL ↓: –2.46 (95% CI: –4.90 to –0.02, fruits & vegetables); HDL ↑: 0.98 (95% CI: 0.18 to 1.79, high-fat dairy); CRP ↑: 0.10 (95% CI: 0.003 to 0.21, western-type); CRP ↑: 0.09 (95% CI: 0.00 to 0.18, high-fat dairy); Adiponectin ↑: 0.04 (95% CI: 0.00 to 0.07, fruits & vegetables). Biomarkers (women): LDL ↓: –2.02 (95% CI: –3.72 to –0.32, fruits & vegetables); Cholesterol ↓: –2.52 (95% CI: –4.61 to –0.42, fruits & vegetables); CRP ↑: 0.10 (95% CI: 0.02 to 0.18, western-type); CRP ↓: –0.09 (95% CI: –0.16 to –0.01, high-fat dairy).
	Malmö	Mandalazi, 2016 [99]	Fiber adherence (both, mainly women)	17 years	HR = 0.85 (95% CI: 0.78–0.92); HR = 0.91 (95% CI: 0.84–0.99)
Sweden	Västerbotten County	Long, 2015 [120]	Composite health score (worst 0-1 vs healthiest) (both, mainly women)	9.9 years	RR = 3.74 (95% CI: 2.50–5.59)
	Scania county	Herzog, 2021 [111]	Individual and combined lifestyle factors (PA, diet, BMI, smoking, alcohol) and genetic risk	Not available	Healthy lifestyle: OR = 0.05 (95% CI: 0.03–0.09), Men = 0.05 (0.02–0.11), Women = 0.06 (0.03–0.13); BMI = 0.12 (0.10–0.15); PA = 0.48 (0.41–0.55); Diet = 0.75 (0.61–0.94); Smoking = 0.73 (0.63–0.83); Alcohol = 0.60 (0.52–0.69); Family history (+) = 0.09 (0.04–0.19); TCF7L2 (TT/TC) = 0.03 (0.01–0.12); HLA low risk = 0.03 (0.01–0.11).
France	Not available	Rajaobelina, 2019 [52]	Obesity, smoking, and low physical activity (women)	18 years	HR = 9.65 (95% CI: 8.57–10.86); HR = 1.67 (95% CI: 1.47–1.90); HR = 1.41 (95% CI: 1.21–1.65)

Country	Region	Author, Year	Exposure Summary	Follow-up time	Effect Estimate (95% CI)
	Not available	Kesse-Guyot, 2020 [100]	Organic food consumption, including plant- and animal-based products (women)	4.06 years	Higher organic food intake women: HR = 0.35 (95% CI: 0.19–0.63), low-income individuals (Q5): HR = 0.20 (95% CI: 0.04–0.94).
	Not available	Dow, 2016 [118]	n-3 PUFA intake ≥1.6 g/day (women)	18 years	HR = 1.26 (95% CI: 1.13–1.41)
	Not available	Kesse-Guyot, 2021 [101]	PNNS guideline adherence (highest quintile) (both, mainly women)	6.7 years	HR = 0.46 (95% CI: 0.35–0.62)
Netherlands	Northern Netherlands	Ming-Jie, 2021 [122]	Dietary pattern healthiness ranked from unhealthiest to healthiest (men and women)	43 months	Women, unhealthiest dietary pattern: OR = 1.57 (95% CI: 1.01–2.44); Men, unhealthiest quintile OR = 1.65 (95% CI: 1.04–2.62).
	Rotterdam, Ommoord	Chen, 2018 [30]	Healthy dietary pattern adherence (both, mainly women)	7.3 years	HR = 0.87 (95% CI: 0.79–0.99); HR = 0.82 (95% CI: 0.73–0.92)

Country	Region	Author, Year	Exposure Summary	Follow-up time	Effect Estimate (95% CI)
Denmark	Eastern Denmark, Copenhagen and Aarhus	Markanti, 2021 [102]	Dietary scores and food group intake (both, mainly women)	15 years	Diet score ≥5: HR = 0.82 (95% CI: 0.69–0.96, women), HR = 0.70 (95% CI: 0.58–0.85, men); Diet score 3–<4: HR = 0.80 (95% CI: 0.74–0.87, men); Diet score 4–<5: HR = 0.71 (95% CI: 0.65–0.78, men); Fruit & vegetables: HR = 0.77 (95% CI: 0.66–0.89, men), HR = 0.79 (95% CI: 0.67–0.94, women); Whole grains: HR = 0.81 (95% CI: 0.71–0.92, men); Low-fat meat & cold meat: HR = 0.86 (95% CI: 0.77–0.95, men), HR = 0.86 (95% CI: 0.77–0.96, women); Saturated fat: HR = 1.24 (95% CI: 1.05–1.47, women); Sugar: HR = 0.76 (95% CI: 0.63–0.91, men), HR = 0.83 (95% CI: 0.70–0.99, women).
	Copenhagen and Aarhus	Lacoppidan, 2015 [103]	Adherence to the Healthy Nordic Food Index and intake of Nordic diet components (e.g., oatmeal, rye bread, root vegetables) (men and women)	15.3 years	Highest adherence: women HR = 0.68 (95% CI: 0.56–0.84), men HR = 0.54 (95% CI: 0.47–0.62). Food items: Women: oatmeal 0.74 (0.67–0.81), root vegetables 0.84 (0.78–0.90); Men: oatmeal 0.75 (0.69–0.82), cabbage 0.89 (0.83–0.95), root vegetables 0.78 (0.73–0.83)
Finland	Kuopio	Tertsunen, 2021 [119]	Healthy Nordic Diet (lowest vs highest score) (men)	19.3 years	HR = 1.35 (95% CI: 1.03–1.76)
	Not available	Montonen, 2005 [31]	Prudent Pattern (High in fruits and vegetables, Conservative Pattern (High in butter, potatoes, whole milk) (men and women)	23 years	Prudent diet: RR = 0.72 (95% CI: 0.53–0.97); women RR = 0.56 (0.38–0.85), age ≥51 RR = 0.51 (0.34–0.77), BMI ≥26.1 RR = 0.68 (0.48–0.97); Conservative diet: RR = 1.49 (1.11–2.00); especially in women RR = 1.49 (1.02–2.19), age ≥51 RR = 1.62 (1.10–2.39), smokers RR = 2.35 (1.17–4.71), BMI ≥26.1 RR = 1.44 (1.03–2.01).

Country	Region	Author, Year	Exposure Summary	Follow-up time	Effect Estimate (95% CI)
Italy	Not available	Mozaffarian, 2007 [104]	Traditional Mediterranean diet adherence (Dietary score 9–10; Dietary score 11–15) (both, mainly men)	3.5 years	HR = 0.76 (95% CI: 0.60–0.97); HR = 0.63 (95% CI: 0.48–0.82)
Multinational	Denmark, France, Germany, Netherlands, Spain, Sweden, UK	The Interact Consortium, 2014 [105]	Prudent and anti-inflammatory dietary patterns (men and women)	Not available	UK= HR = 0.67 (95% CI: 0.57–0.79); Germany= HR = 0.82 (95% CI: 0.76–0.89); Denmark and Spain= HR range = 0.88 (95% CI: 0.83–0.94); Spain 0.90 (95% CI: 0.82–0.98)
		Buijsse, 2015 [106]	Vegetable oils, margarine, nuts (both, mainly women)	12.3 years	Highest consumers of margarine HR = 1.12 (95% CI: 0.95–1.31); across consumption groups HR = 0.88 (95% CI: 0.75–1.03)
Multinational	Denmark, France, Germany, Italy, Netherlands, Spain, Sweden, UK	Patel, 2012 [107]	Fatty fish consumption (both, mainly women)	3.99 million person-years	HR = 0.87 (95% CI: 0.78–0.97)
		The InterAct Consortium, 2013 [108]	Dietary energy density (DED)	3.99 million person-years	HR = 0.88 (95% CI: 0.79–0.99) (not conclusive results overall)

[a] Abbreviations. UK= United Kingdom; HR= Hazard ratios; OR= Odds ratios; RR= Relative risks; RD= risk difference; IRR= Incidence rate ratios; BMI= Body Mass Index; IFG= Impaired Fasting Glucose; IGT= Impaired Glucose Tolerance; SFA= Saturated Fatty Acids; UPF= Ultra-Processed Foods; ASB= Artificially Sweetened Beverages; PREDIMED= Prevención con Dieta Mediterránea; PNNS= Programme National Nutrition Santé; PUFA= Polyunsaturated Fatty Acids.
